# Prognostic and Associative Significance of Malnutrition in Sarcopenia: A Systematic Review and Meta-Analysis

**DOI:** 10.1016/j.advnut.2025.100428

**Published:** 2025-04-11

**Authors:** Konstantinos Prokopidis, Giuseppe Dario Testa, Christoforos D Giannaki, Pinelopi Stavrinou, Eirini Kelaiditi, Emiel O Hoogendijk, Nicola Veronese

**Affiliations:** 1 Department of Musculoskeletal and Ageing Science, Institute of Life Course and Medical Sciences, University of Liverpool, Liverpool, United Kingdom; 2 Department of Geriatric and Intensive Care Medicine, Careggi Hospital, University of Florence, Florence, Italy; 3 Department of Life Sciences, School of Life and Health Sciences, University of Nicosia, Nicosia, Cyprus; 4 Faculty of Sport, Allied Health and Performance Science, St Mary’s University, Twickenham, United Kingdom; 5 Department of General Practice, Amsterdam Public Health research institute, VU University Medical Center, Amsterdam, The Netherlands; 6 Department of Epidemiology & Data Science, Amsterdam Public Health research institute, VU University Medical Center, Amsterdam, The Netherlands; 7 Department of Health Promotion, Mother Child Care, Internal Medicine and Medical Specialties, University of Palermo, Palermo, Italy

**Keywords:** malnutrition, sarcopenia, mini nutritional assessment, mortality, ageing

## Abstract

Malnutrition is a common phenomenon, particularly in those at an increased risk of muscle mass and function losses. In this systematic review and meta-analysis, we aimed to explore the association of malnutrition with sarcopenia in middle-aged and older adults and the prognostic association of malnutrition and sarcopenia compared with sarcopenia alone on all-cause mortality. PubMed, Scopus, Web of Science, and Cochrane Library were searched from inception until January 2024. A meta-analysis using a random-effect model was employed, utilizing the Mini Nutritional Assessment malnutrition tool as a continuous and categorical variable. The study protocol was registered in the International Prospective Register of Systematic Reviews (CRD42024501521). Malnutrition was significantly associated with a greater risk of sarcopenia [continuous: *k* = 12, odds ratio (OR): 1.38, 95% confidence interval (CI): 1.18, 1.61, *I*^2^ = 94.8%, *P* < 0.01; categorical: *k* = 37, OR: 2.99, 95% CI: 2.26, 3.96, *I*^2^ = 78.3%, *P* < 0.01]. Sarcopenia and malnutrition were associated with a higher risk of mortality compared with sarcopenia alone (*k* = 5, hazard ratio: 4.04, 95% CI: 1.36, 11.94, *I*^2^ = 92.8%, *P* < 0.01). Metaregression showed age, sex, and number of adjustments did not explain heterogeneity among studies. The included studies had a moderate risk of bias. Malnutrition is associated with higher odds of sarcopenia and their combined presence is a better predictor of all-cause mortality compared with sarcopenia alone, further highlighting the importance of applying interventions to counteract these two closely related phenomena.

**Statement of significance**

This study quantifies the increased risk of sarcopenia associated with malnutrition and highlights the combined prognostic association of both conditions on mortality. Our analysis explores the heterogeneity across multiple studies and confirms that traditional factors such as age and sex do not explain the observed variability, which may offer new insights into the relationship between malnutrition and sarcopenia.

## Introduction

Life expectancy is projected to increase, and the number of people aged 65 and over will become a much larger share of the population [1]. Parallel with the continued increases in longevity, the prevalence of various geriatric syndromes/diseases is expected to grow [2,3]. One of the most common is sarcopenia which is characterized by low muscle mass and function, beginning around the fourth decade of life with an accelerated loss as age increases, especially in very old age [4].

Since 2016, sarcopenia has been recognized as an independent condition according to the International Classification of Disease, Tenth Revision, Clinical Modification [5]. However, despite the seriousness of this disease, among operational definitions and diagnostics, sarcopenia lacks a consensus [6], with the global prevalence of sarcopenia varying considerably according to the classification used [7,8]. The European Working Group on Sarcopenia in Older People (EWGSOP2), in its revised consensus, focuses on low muscle strength as a key characteristic of sarcopenia, uses detection of low muscle quantity and quality to confirm the sarcopenia diagnosis, and identifies poor physical performance as indicative of severe sarcopenia [9]. The Sarcopenia Definition and Outcomes Consortium (SDOC) defined sarcopenia using muscle strength and function without the inclusion of muscle mass as part of their definition of sarcopenia [10]. However, more recently, the Global Leadership Initiative in Sarcopenia concluded that low muscle mass, strength, and muscle-specific strength are considered components of sarcopenia, whereas impaired physical performance is viewed as an outcome rather than a component [11].

Nevertheless, previous studies using different definitions of sarcopenia have reported its association with negative consequences, such as reduced health-related quality of life [12], functional decline [13], hospitalizations [14], and all-cause mortality, making sarcopenia an emerging public health priority.

The complex and multifactorial etiology of sarcopenia may be explained by factors such as genetic heritability, lower nutritional status, physical inactivity, hormonal changes, skeletal muscle insulin resistance, and changes in circulating proinflammatory cytokines [15]. Among them, malnutrition can be a major causal component in the development of sarcopenia. The established Global Leadership Initiative on Malnutrition (GLIM) operational diagnostic criteria consist of 3 phenotypic (weight loss, low BMI, and reduced muscle mass) and 2 etiologic criteria (reduced food intake or its assimilation and inflammation) [16]. Recently, malnutrition has attracted increasing scientific attention as it has been associated with physical decline, which has wide-ranging acute implications for activities of daily living and quality of life [17], and it has been associated with increased mortality [18]. Hence, it comes as no surprise that the scientific community emphasizes the importance of conducting nutritional screenings for older adults who might need nutritional assistance, aiming to prevent premature death [19].

The role of malnutrition as a contributing factor for the onset of sarcopenia has been redefined recently, and malnutrition is considered a key contributing factor for skeletal muscle loss. There are several ways in which malnutrition can lead to sarcopenia. Malnutrition can result in weight loss, protein deficiency, and micronutrient deficiency (that is, deficiency in vitamin D, calcium, and magnesium), which are vital elements of muscle function and hormonal imbalances that favor muscle loss (that is, increased secretion of cortisol) and inflammation [20]. Interestingly, high levels of inflammation contribute to the development of malnutrition through associated anorexia and decreased food intake although elevating resting energy expenditure, resulting in increased muscle catabolism [16]. People with malnutrition often exhibit poor physical performance and physical inactivity [21], whereby the latter can contribute to muscle atrophy and further exacerbate sarcopenia [22].

The condition known as “malnutrition-sarcopenia syndrome” (MSS) refers to the coexistence of both sarcopenia and malnutrition. Interestingly, in a cohort among hospitalized older people, individuals with MSS were found to experience the worst survival curve compared with individuals with sarcopenia alone, malnutrition alone, or normal nutrition [23].

The objectives of the present meta-analysis were to address the following inquiries in middle-aged and older people: *1*) does malnutrition increase the odds of sarcopenia?; *2*) does the combination of malnutrition and sarcopenia increase risk of all-cause mortality compared with sarcopenia alone?

## Methods

The 2020 PRISMA guidelines were followed to perform this systematic review and meta-analysis [24]. The protocol has been registered in the PROSPERO (CRD42024501521).

### Search strategy

From inception until January 2024, PubMed, Scopus, Web of Science, and Cochrane Library were searched by 2 investigators. The employed key search terms can be found in Supplemental Table 1. Two reviewers (GDT, KP) independently screened the title/abstracts/full texts by applying the required eligibility criteria for inclusion. A third researcher (NV) was available in case of disagreement. Endnote 20.0 was used for title/abstract screening, whereas no software was used for full-text screening.

### Inclusion and exclusion criteria

Studies were included based on the following criteria: *1*) data from observational studies; *2*) adults with mean age >50 y irrespective of health status; *3*) definition of sarcopenia based on definitions established by different groups such as the EWGSOP, Asian Working Group for Sarcopenia (AWGS), SDOC, and Foundation for the National Institute of Health (FNIH); *4*) definition of malnutrition based on consensus from all authors [for example, Mini Nutritional Assessment (MNA) score (either long or short-form), the Malnutrition Universal Screening Tool (MUST), the GLIM criteria for the diagnosis of malnutrition, Controlling nutritional status score (CONUT), the Geriatric Nutritional Risk Index (GNRI), the Prognostic Nutritional Index (PNI), the Patient-Generated Subjective Global Assessment (PG-SGA), and/or the Nutrition Risk Screening 2002 (NRS-2002) score]. Published articles were excluded if they: *1*) were reviews, letters, in vivo or in vitro experiments, commentaries, or posters; *2*) were not published as a full text and in English; *3*) reported the definition of sarcopenia using only 1 method of assessment (for example, handgrip strength, body composition); *4*) used tools to screen and not diagnose sarcopenia (for example, the strength, assistance with walking, rising from a chair, climbing stairs, and falls (SARC-F) questionnaire); *5*) data could not be meta-analyzed (for example, relative risk or hazard ratio (HR) for the association between sarcopenia and malnutrition); *6*) combined data not differentiating sarcopenia from pre-sarcopenia; and *7*) reported data from cohorts already reported in another included study (in this case we considered the largest in terms of sample size).

More specifically, applying the Participants Exposure Comparator Outcomes Study Design (PECOS) criteria, we included the following for each objective:

1) Does malnutrition increase the odds of sarcopenia?

P) Adults with a mean age >50 y irrespective of health status

E) Malnutrition defined via the MNA score (either long or short-form), the MUST, the GLIM criteria for the diagnosis of malnutrition, CONUT, the GNRI, the PNI, the PG-SGA, and/or the NRS-2002 score

C) Adults without malnutrition

O) Odds ratio (OR) of sarcopenia

S) Observational studies

2) Does the combination of malnutrition and sarcopenia increase risk of all-cause mortality compared with sarcopenia alone?

P) Adults with a mean age >50 y irrespective of health status

E) Malnutrition defined via the MNA score (either long or short-form), the MUST, the GLIM criteria for the diagnosis of malnutrition, CONUT, the GNRI, the PNI, the PG-SGA, and/or the NRS-2002 score combined with sarcopenia defined via the EWGSOP, AWGS, SDOC, and FNIH

C) Adults with sarcopenia, but without malnutrition

O) HR of all-cause mortality

S) Observational studies using a longitudinal design

### Data extraction and risk of bias

Two authors (GDT, KP) extracted data independently, which included the name of the first author, year of publication, country of origin, study design, the definition of sarcopenia and malnutrition, patient characteristics (sample size, age, sex, BMI, health status), body composition assessment tool, and reported comorbidities. Disagreements between authors were resolved by a third investigator (NV).

About outcomes, the odds of malnutrition with sarcopenia were extracted using ORs alongside their 95% confidence intervals (CIs) from multivariate analyses, using the most adjusted model where available for both continuous and categorical data. Similarly, when we attempted to predict the hazardous risk of malnutrition combined with sarcopenia compared with sarcopenia alone in increasing risk of all-cause mortality, we extracted data using the most adjusted HRs for each model for both continuous and categorical data.

For the objective “Does malnutrition increase the odds of sarcopenia?” the references used for malnutrition definition, which are included in our analysis are presented in Supplemental Table 2.

For the objective “Does the combination of malnutrition and sarcopenia increase risk of all-cause mortality compared to sarcopenia alone?” the references used for malnutrition definition, which are included in our analysis are presented in Supplemental Table 3.

The quality of the included studies was evaluated using the Newcastle Ottawa Scale (NOS) by 2 authors independently. Any disagreements were resolved along with a third member of the team. NOS assigns a maximum of 9 points according to 3 parameters: selection, comparability, and outcome. Two authors evaluated risk of bias in the included studies, which were considered as high (<5 points), moderate (6–7 points), or low (8–9 points) [25].

### Data analysis

The meta-analysis was performed using STATA version 13.0 (StataCorp). We calculated OR and HRs with their 95% CIs using a random-effect model. Considering that malnutrition data were extracted as continuous and categorical, we employed meta-analyses for both. Heterogeneity across studies was assessed by *I*^2^ and *χ*^2^. In case of increased heterogeneity (*I*^2^ ≥ 50%) and for outcomes consisting of ∼10 studies, metaregression analyses were conducted based on age, sex, follow-up months, and number of adjustments as well as setting, criteria to define malnutrition and sarcopenia. Publication bias was assessed by Egger’s test and visually presented through funnel plots [26]. When publication bias was detected, the trim-and-fill analysis was used to account for it [27]. Regarding the imputation of values pertinent to malnutrition, we used values that represented malnutrition based on the definition applied by the authors of the included studies. Finally, a *P* value < 0.05 was considered statistically significant.

## Results

### Search results

**FIGURE 1:**
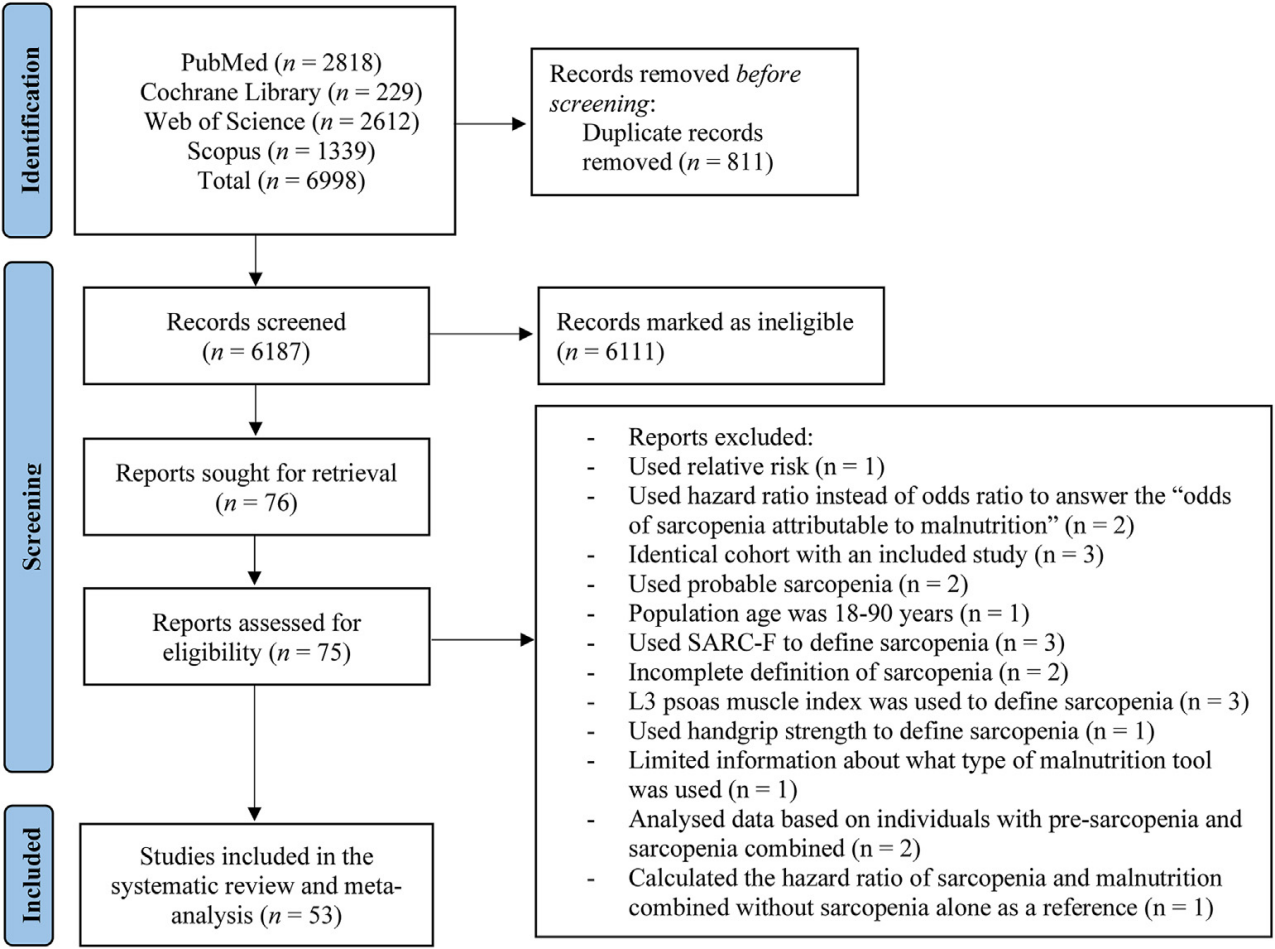
Flowchart of the search methods. SARC-F, strength assistance with walking rising from a chair climbing stairs and falls.

The initial literature search showed 6998 publications. Following the exclusion of duplicates and abstracts, 75 full texts were identified as eligible for inclusion in this study. Of these 75 studies, 53 were finally deemed eligible for inclusion (Figure 1). Overall, 48 studies evaluated the odds of sarcopenia attributable to malnutrition, whereas 5 studies examined the prognostic association of malnutrition and sarcopenia compared with sarcopenia alone for mortality. Excluded studies are shown in Supplemental File 1. Supplemental Table 2 shows the main characteristics of the studies indicating that malnutrition increases the odds of sarcopenia: we were able to find 48 studies dealing with this topic for a total of 23,269 participants. The mean age was 73 y, while 60.1% were women. As better detailed in Supplemental Table 2, the tools used for malnutrition and sarcopenia were heterogeneous.

Furthermore, Supplemental Table 3 shows studies used to assess the prognostic association of malnutrition and sarcopenia compared with sarcopenia alone on mortality. Overall, the 5 studies included a total of 1579 participants, mainly from hospitals. All the studies included a population aging a mean >65 y, except 1, although the percentage of women was, in total, 50%. The mean follow-up period was ∼2 y. In all studies, except 1, the diagnosis of sarcopenia was made using the EWGSOP2 criteria. Finally, as a tool to identify/screen malnutrition, there was a balance between MNA and SGA, with 3 studies using these tools, respectively.

### Odds of sarcopenia due to malnutrition

**FIGURE 2:**
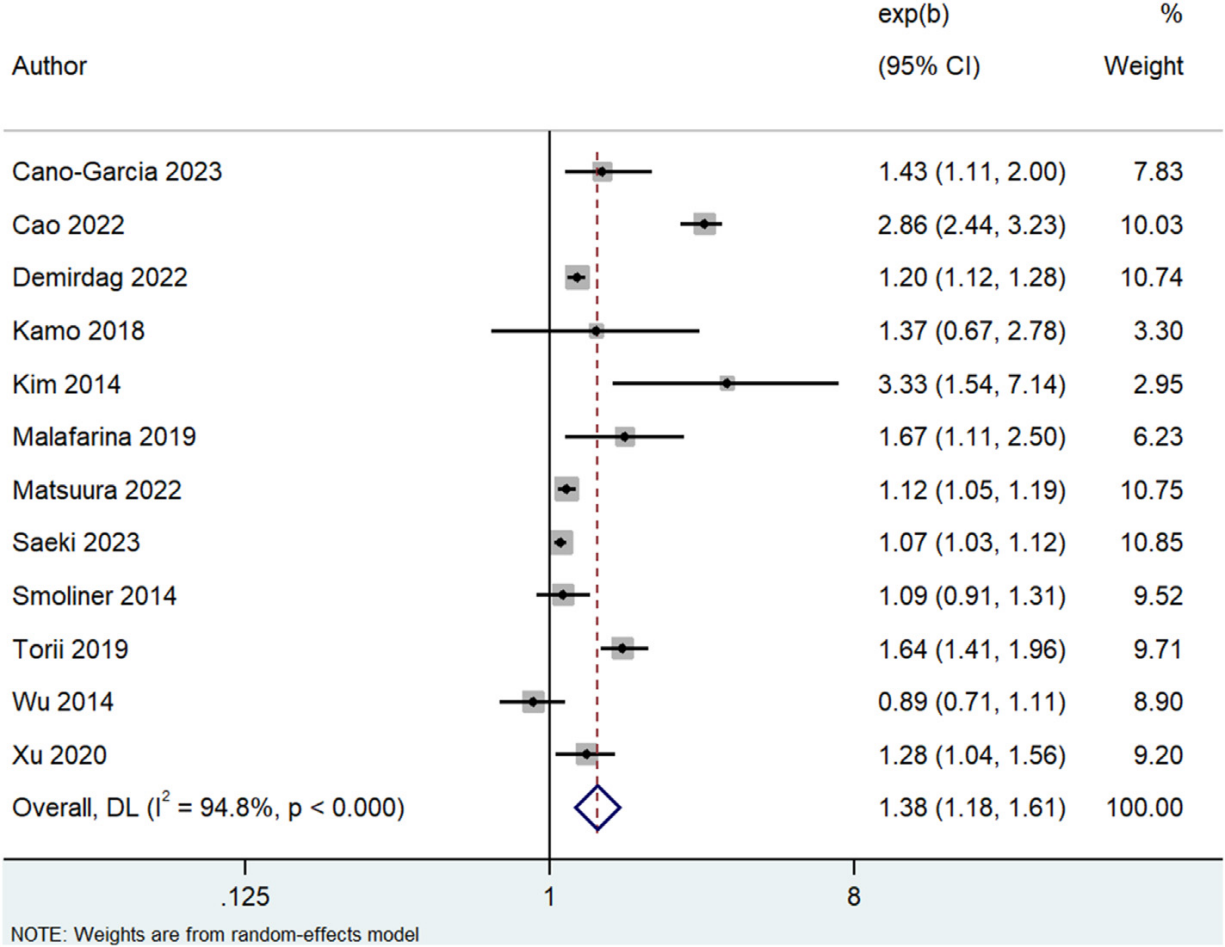
Odds of sarcopenia due to malnutrition (continuous variable). CI, confidence interval; DL, DerSimonian and Laird.

Using malnutrition as a continuous variable, it was significantly associated with greater odds of sarcopenia (*k* = 12, OR: 1.38, 95% CI: 1.18, 1.61, *I*^2^ = 94.8%, *P* < 0.01) (Figure 2).

No publication bias was observed (Supplemental Table 4), although age, sex, number of adjustments, and definition of malnutrition tool or sarcopenia definition did not explain the increased heterogeneity (Supplemental Table 5).

**FIGURE 3:**
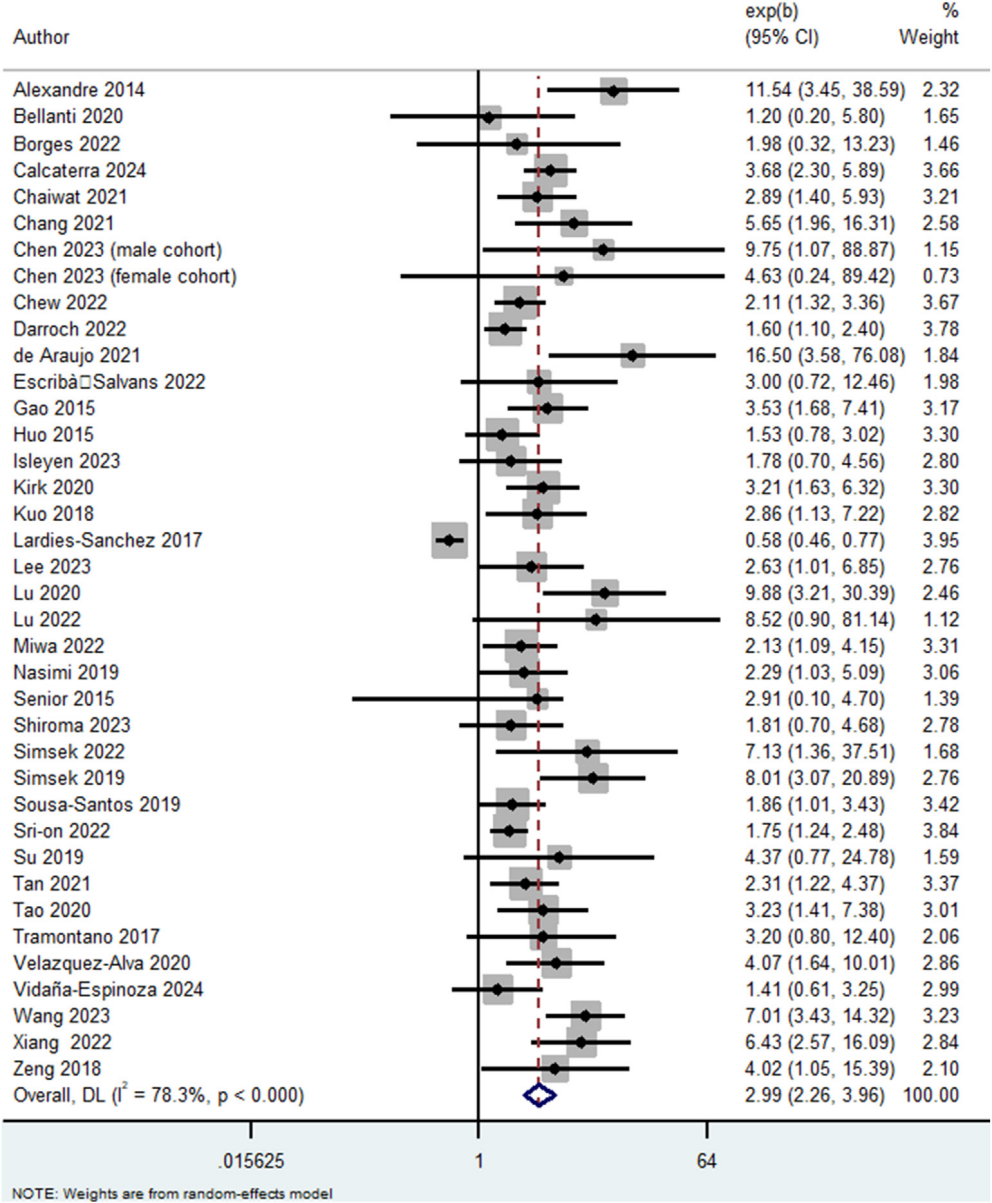
Odds of sarcopenia due to malnutrition (categorical variable). CI, confidence interval; DL, DerSimonian and Laird.

When malnutrition was used as a categorical variable, we similarly found a significant association with a higher risk of sarcopenia (*k* = 38, OR: 2.99, 95% CI: 2.26, 3.96, *I*^2^ = 78.3%, *P* < 0.01) (Figure 3).

Publication bias was detected (*P* < 0.01), and using the trim-and-fill method, OR was recalculated to 2.23 with a 95% CI (1.74, 2.88) (Supplemental Table 6). Age, sex, and number of adjustments did not explain the increased heterogeneity (Supplemental Table 7).

### Prognostic association of the combination of sarcopenia and malnutrition on all-cause mortality compared with sarcopenia alone

**FIGURE 4:**
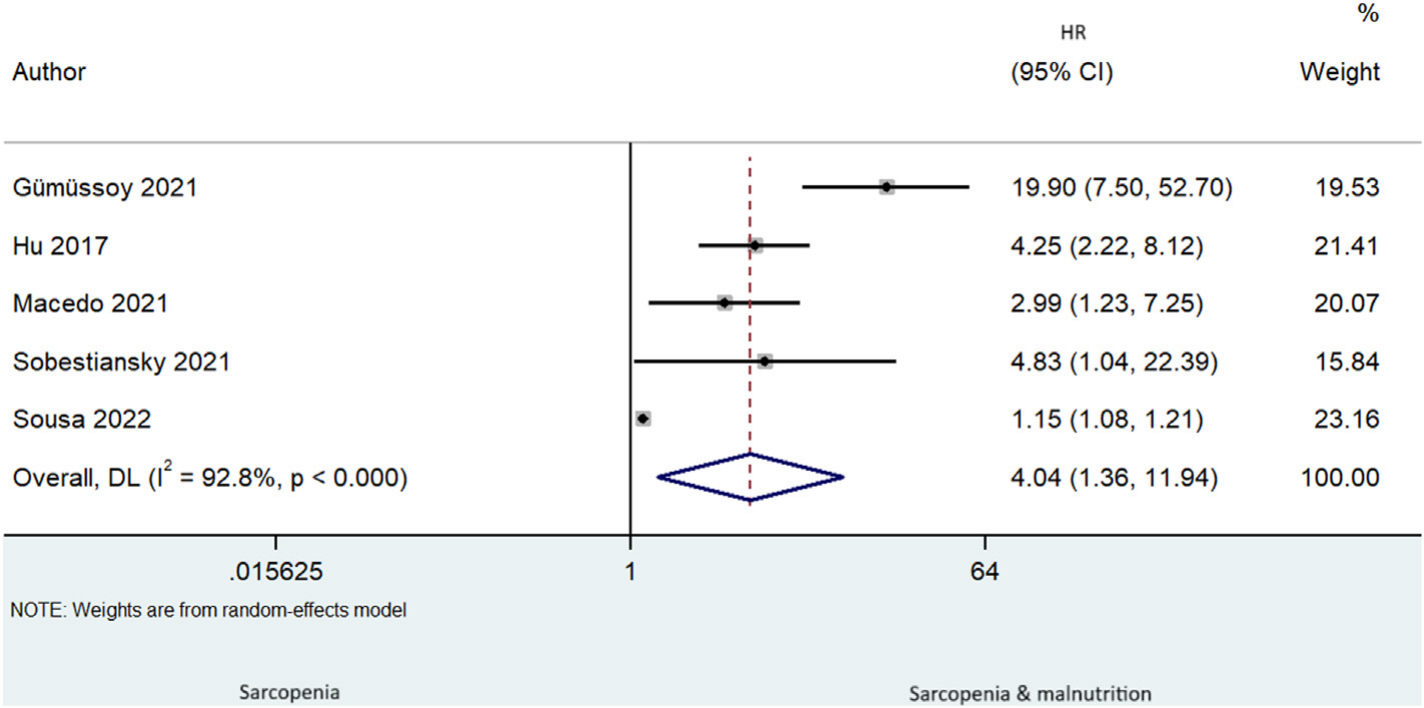
Sarcopenia and malnutrition compared with sarcopenia alone as a significant prognostic factor of mortality. CI, confidence interval; DL, DerSimonian and Laird.

The combination of sarcopenia and malnutrition compared with sarcopenia alone was a significant prognostic factor of all-cause mortality (*k* = 5, HR: 4.04, 95% CI: 1.36, 11.94, *I*^2^ = 92.8%, *P* < 0.01) (Figure 4).

Publication bias was detected (*P* = 0.02); however, the recalculated HR through trim-and-fill remained unchanged (Supplemental Table 8). Age, sex, follow-up months, and number of adjustments, setting, criteria to define sarcopenia and malnutrition did not explain the increased heterogeneity (Supplemental Table 9). Categorical and continuous funnel plots before and after trim-and-fill are presented in Supplemental Figures 1–4.

### Risk of bias

The overall risk of bias assessment of prospective studies was considered moderate (Supplemental Table 10). From the cross-sectional studies, 3 studies were considered of having an increased risk of bias [[28], [29], [30]], whereas overall, a moderate risk of bias was demonstrated. The complete score of each domain is presented in Supplemental Table 11.

## Discussion

In this systematic review and meta-analysis, we found that malnutrition is associated with increased odds of sarcopenia, whereas malnutrition and sarcopenia combined may significantly increase incident all-cause mortality rates compared with sarcopenia alone. Age, sex, and number of adjustments did not impact the analyses, nor the observed publication bias.

Numerous studies have demonstrated a strong association between malnutrition and sarcopenia, with various underlying parameters contributing to this relationship. For example, Beaudart et al. [28] reported that malnutrition was linked to a nearly fourfold increased risk of developing sarcopenia over a 4-y follow-up period. A key factor may be attributed to the reduction in muscle mass and strength due to malnutrition, for which, malnourished individuals have been shown to exhibit lower muscle thickness and cross-sectional area [29]. Additionally, inadequate energy and protein intake, a common feature of malnutrition, further impairs muscle protein synthesis, contributing to progressive muscle loss and the onset of sarcopenia [30,31]. Moreover, lower physical activity or being sedentary, frequently observed in malnourished individuals, compounds this issue by accelerating muscle atrophy and functional decline [32]. These interconnected factors highlight the importance of addressing malnutrition as a critical component in the prevention and management of sarcopenia.

The combined impact of malnutrition and sarcopenia on increased all-cause mortality rates can be explained via multiple parameters. As stated previously, malnutrition described by inadequate intake of protein and energy intakes may lead to reductions in muscle protein synthesis and even vitamin and mineral inadequacy, which chronically, may be essential for muscle preservation. This deficiency exacerbates muscle wasting, increasing risk of functional decline, increased risk of frailty and dependency [21], and, ultimately, mortality [33,34]. Malnutrition has also been linked to extended hospital stays and higher readmission rates [35,36], which are hallmarks of muscle disuse, leading to acute sarcopenia and worsened rehabilitation and recovery rates [37]. These parameters highlight the critical need for early detection and targeted interventions to mitigate the adverse outcomes associated with these 2 conditions.

Our findings highlight further the critical importance of nourishment in older age, particularly in those at risk of sarcopenia. A previous meta-analysis showed a substantial overlap among frailty, sarcopenia, and malnutrition in older hospitalized adults [38], populations with several concomitant comorbidities, which may exacerbate mortality risk. Interestingly, reduced rates of home discharge, and accelerating rates of health service use and physical dysfunction, have been proposed as contributing factors in this interplay in a recent study examining older adults undergoing rehabilitation [39]. Furthermore, appetite loss occurring during aging and under multiple conditions inducing cachexia may amplify sarcopenia and subsequent mortality risk, regardless of the healthcare setting [40]. These results may be extended to individual measures of sarcopenia, considering that impaired handgrip strength, gait speed, time and go test, and short physical performance battery are all linked to a greater risk of malnutrition [21]. Although there is a pressing need to utilize interventions that may counteract malnutrition to prevent/manage sarcopenia, the malnutrition tool applied for diagnosis should be taken into account. Therefore, an established tool in each setting may ensure greater reliability and consistency [41]. In this meta-analysis, the MNA was primarily employed to calculate the effect estimates in the association between malnutrition and sarcopenia, given the lack of studies and inconsistencies across statistical methods among other diagnostic tools.

### Strength and limitations

In this study, an examination of multiple malnutrition tools was employed, which helped review the currently utilized tools to assess their link with sarcopenia. Our study, however, was prone to limitations. For instance, we could not conduct meta-analyses and quantify our results based on all malnutrition tools, separately, due to a lack of studies; hence, we were only able to observe associations based on MNA or a combination of MNA and SGA assessments. Additionally, although the prognostic factor of MNA was significant in combination with sarcopenia, it was merged with SGA which may have underestimated its prognostic association on mortality. Furthermore, a meta-analysis on individuals with different reported comorbidities was not conducted, which may have alleviated the robustness of the results due to higher heterogeneity and confounding, in terms of its application to the broader population. Lastly, it is worth noting that our analyses were based on different definitions of sarcopenia and malnutrition tools, displaying increased heterogeneity among studies.

In conclusion, in this systematic review and meta-analysis, we found that malnutrition is significantly associated with a higher risk of sarcopenia. Additionally, we also found that all-cause mortality is exacerbated in individuals with combined malnutrition and sarcopenia compared with sarcopenia alone. These results highlight the importance of well nourishment in older adults, particularly those at risk of sarcopenia. Our study showed an increased application of the MNA as opposed to other diagnostic tools, emphasizing the need for consistency and feasibility. Despite our reliance on a single tool, our findings further reinforce the need for targeted interventions to counteract malnutrition and manage sarcopenia.

## Author contributions

The authors’ responsibilities were as follows – KP: designed the study; KP, CDG, PS: wrote the manuscript; GDT, NV: conducted the analyses; EK, EOH, NV: revised the manuscript; and all authors: read and approved the final manuscript.

## Data availability

Data are available on request.

## Funding

The authors reported no funding received for this study.

## Conflict of interest

The authors report no conflicts of interest.
