# Supplementary material for: Prognostic and Associative Significance of Malnutrition in Sarcopenia: A Systematic Review and Meta-Analysis
Source: Adv Nutr. 2025 Apr 11;16(5):100428. doi: 10.1016/j.advnut.2025.100428 (PMC12099873; doi:10.1016/j.advnut.2025.100428)
Supplement: Multimedia component 1 [file mmc1.docx]

**Prognostic and associative significance of malnutrition in sarcopenia: a systematic review and meta-analysis**

Konstantinos Prokopidis

**Supplementary Materials**

**Supplementary Table S1.**Search terms employed in the screening based on title, abstract, and keywords in the literature search.

| **Database** | **Search terms** |
| --- | --- |
|  |  |
| PubMed | sarcopeni* AND (malnutrition OR mini nutritional assessment OR Subjective Global Assessment of Malnutrition OR  Geriatric Nutritional Risk Index OR Malnutrition Universal Screening Tool OR "GNRI" OR Controlling nutritional status score OR  “CONUT” OR Nutrition Risk Screening 2002 OR “NRS-2002” OR Global Leadership Initiative on Malnutrition OR "GLIM" OR  Patient-Generated Subjective Global Assessment OR "PG-SGA" OR Prognostic Nutritional Index OR “PNI”) |
| Cochrane Library | sarcopeni* AND (malnutrition OR mini nutritional assessment OR Subjective Global Assessment of Malnutrition OR  Geriatric Nutritional Risk Index OR Malnutrition Universal Screening Tool OR "GNRI" OR Controlling nutritional status score OR  “CONUT” OR Nutrition Risk Screening 2002 OR “NRS-2002” OR Global Leadership Initiative on Malnutrition OR "GLIM" OR  Patient-Generated Subjective Global Assessment OR "PG-SGA" OR Prognostic Nutritional Index OR “PNI”) |
| Web of Science | sarcopeni* AND (malnutrition OR mini nutritional assessment OR Subjective Global Assessment of Malnutrition OR  Geriatric Nutritional Risk Index OR Malnutrition Universal Screening Tool OR "GNRI" OR Controlling nutritional status score OR  “CONUT” OR Nutrition Risk Screening 2002 OR “NRS-2002” OR Global Leadership Initiative on Malnutrition OR "GLIM" OR  Patient-Generated Subjective Global Assessment OR "PG-SGA" OR Prognostic Nutritional Index OR “PNI”) |
| Scopus | sarcopeni* AND ( malnutrition OR mini AND nutritional AND assessment OR "GNRI" OR “CONUT” OR “MUST” OR  "GLIM" OR "PG-SGA" OR “NRS-2002” OR “PNI”) |

**Supplementary Table 2.** Studies including odds ratio of sarcopenia due to malnutrition.

| **Author** | **Year** | **Country** | **Study design** | **Source of Cohort** | **Sample Size** | **Mean Age (SD)** | **Females %** | **Follow-up Measurement (months)** | **Sarcopenia Definition** | **Muscle Mass Assessment Tool** | **Sarcopenia Indices** | **Malnutrition tool** | **Malnutrition Definition** | **Odds ratio of Sarcopenia with Malnutrition (95% CI)** | **Type of Adjustments** | **N° of Adjustments** |
| --- | --- | --- | --- | --- | --- | --- | --- | --- | --- | --- | --- | --- | --- | --- | --- | --- |
| Alexandre | 2014(1) | Brazil | Cross-sectional | Community-dwelling | 1149 | 69.6 (0.6) | 62 | - | EWGSOP1 | Lee equation | ASM/h2, HGS, GS | MNA-lf | <17 | 11.54 (3.45-38.59) | Age, gender, income, marital status, Schooling, smoking, lifestyle, MMSE | 8 |
| Bellanti | 2020(2) | Italy | Prospective | Hospitalized | 152 | 78.25 (7.5) | 42 | - | EWGSOP2 | BIA | ASM/h2, HGS, GS | MUST, SGA, GLIM, NRS-2002 | MUST≥2  SGA>3  GLIM one phenotypic criterion and one etiologic criterion,  NRS-2002 >2 | GLIM: 2.7 (1.4 - 4.9);  NRS-2002 1.2. (0.2 - 5.8);  MUST (high risk): 2.5 (1.3 - 3.6);  SGA: 2.7 (0.2 - 9.4) | Age, gender, and education | 3 |
| Cao | 2022(3) | China | Cross-sectional | Outpatients | 1505 | 77.02 (6.92) | 57 | - | AWGS 2019 | BIA | SMI, HGS, GS | MNA-sf | <11 | 0.35 (0.31-0.41) | Age, sex, education level, household income, alcohol drinking, smoking, chronic diseases, and physical activity score | 7 |
| Chaiwat | 2021(4) | Thailand | Prospective | Surgical Cancer | 251 | 71.6 (7.6) | 42 | 12 | EWGSOP2 | BIA | ASM/h2, HGS, GS | MNA-sf | ≤7 | 2.89 (1.4-5.93) | Gender, ASA Classification, diabetes mellitus, hypertension,  dyslipidemia, chronic kidney disease/end-stage renal disease, current smoker, alcohol consumption, waiting time for  surgery, and infection. | 10 |
| Chang | 2021(5) | Taiwan | Cross-sectional | Daycare Centres | 170 | 81.6 (6.8) | 75 | - | AWGS 2019 | BIA | SMI, HGS, GS | MNA-sf | <11 | 5.65 (1.96-16.31) | Demographic  characteristics, risk of malnutrition, 5-times sit-to-stand test, 4 m GS. | 4 |
| Chen | 2023(6) | Germany | Cross-sectional | Community-dwelling | 80 | 81.3 (6.4) | 56 | - | EWGSOP2 | BIA | ASM/h2, HGS | MNA-lf | <17 | Males: 9.75 (1.07-88.87)  Females: 4.63 (0.24 - 89.42) | - | 0 |
| Chew | 2022(7) | Singapore | Cross-sectional | Community-dwelling | 811 | 74.0 ± 0.3 | 53 | - | AWGS 2019 | BIA | SMI, HGS, CST | MUST | ≥2 | 2.11 (1.32 - 3.36) | Age, gender, ethnicity, education  level, smoking and drinking status,  calf circumference, bone mass, and PASE score | 8 |
| Darroch | 2022(8) | New Zealand | Cross-sectional | Aged Care | 91 | 86 (8.3) | 64 | - | EWGSOP2 | BIA | ASM/h2, HGS, GS | MNA-sf | ≤7 | 1.6 (1.1-2.4) | - | 6 |
| de Araujo | 2021(9) | Brazil | Prospective | COPD | 208 | 67.6 (10.1) | 54 | - | EWGSOP2 | CC | HGS | SGA | >3 | 16.5 (3.58 - 76.08) | Age, gender, ethnicity, older age, CCI, CRP, education, ex-smoker, exacerbation, mMRC, FEV, FVC | 12 |
| Escribà-Salvans | 2022(10) | Spain | Cross-sectional | Nursing Home | 104 | 84.6 (7.8) | 85 | - | EWGSOP2 | BIA | ASM, HGS, GS | MNA-lf | ≤23.5 | 3.00 (0.72-12.46) | - | 0 |
| Gao | 2015(11) | China | Cross-sectional | Community-dwelling | 612 | 70.6 (6.7) | 58 | - | AWGS 2014 | CC | HGS, GS | MNA-sf | ≤7 | 3.53 (1.68-7.41) | Age, gender, education level, marital status, monthly  income, smoking status, alcohol-consuming status, physical activity status, comorbidities, the number of diseases, the number of medications, BMI, , depression, ADL disability, IADL disability, and cognitive impairment | 17 |
| Isleyen | 2023(12) | Turkey | Cross-sectional | Cancer | 180 | 73 (5.6) | 50 | - | EWGSOP1 | CC | HGS | MNA-lf | <17 | 1.78 (0.7-4.56) | Age, sex, BMI | 3 |
| Kamo | 2018(13) | Japan | Cross-sectional | Nursing Home | 250 | 86.4 (7.7) | 82 | - | AWGS 2014 | NIRS | SMI, HGS, GS | MNA | MNA analysed as continuous variable | 0.73 (0.36-1.49) | - | 0 |
| Kim | 2014(14) | Korea | Cross-sectional | End-stage renal disease | 95 | 63.9 (10) | 43 | - | EWGSOP1 | BIA | Lean Tissue Index, HGS | SGA | SGA analysed as continuous variable (per unit decrease) | 0.3 (0.14 - 0.65) | Age, gender, BMI,  diabetes | 4 |
| Kirk | 2020(15) | Australia | Cross-sectional | Community-dwelling | 356 | 78.3 (7.44) | 75 | - | SDOC | DXA | ALM/h2, HGS, GS | MNA-lf | ≤23.5 | 3.21 (1.63-6.32) | Age, sex, body mass index, bone mineral density, comorbidity index, vitamin D, and parathyroid hormone | 7 |
| Kuo | 2018(16) | Taiwan | Cross-sectional | Community-dwelling | 731 | 74.5 (5.3) | 47 | - | AWGS 2014 | DXA | SMI, HGS, GS | MNA-lf | ≤23.5 | 2.86 (1.13-7.22) | - | 0 |
| Lardies-Sanchez | 2017(17) | Spain | Cross-sectional | Nursing Home | 339 | 84.9 (7.6) | 64 | - | EWGSOP1 | BIA | ASM/h2, GS, HGS | MNA-lf | ≤17 | 0.58 (0.46-0.77) | Age and sex | 2 |
| Lee | 2023(18) | Taiwan | Longitudinal | Cirrhosis | 45 | 59.8 (10) | 16 | 12 | AWGS 2019 | DXA | ASM/h2, HGS, 6MWD | SGA | >3 | 2.625 (1.006 - 6.847) | - | 0 |
| Malafarina | 2019(19) | Spain | Prospective | Hip Fracture | 116 | 85.2 (6.3) | 92 | 84 | EWGSOP2 | BIA | SMI, HGS, GS | MNA | MNA score analysed as continuous variable | 0.6 (0.4-0.9) | Age, and centre | 2 |
| Matsuura | 2022(20) | Japan | Retrospective | T2D | 234 | 75.71 (7.54) | 59 | 12 | AWGS 2019 | BIA | SMI, HGS | GNRI | GNRI analysed as continuous variable | 0.892 (0.839-0.948) | Age, diabetes duration, dyslipidemia,  nephropathy, and glinide use | 5 |
| Miwa | 2022(21) | Japan | Retrospective | Chronic Liver Disease | 406 | 74 (9.1) | 32 | 24 | Japan Society of Hepatology | CT | SMI, HGS | SGA | >3 | 2.13 (1.09 - 4.15) | Age, sex, etiology of CLD, hepatocellular carcinoma, and Child–Pugh score, sample size, number of events, and confounders | 8 |
| Nasimi | 2019(22) | Iran | Cross-sectional | Community-dwelling | 501 | 70.3 (4.6) | 49 | - | AWGS 2014 | BIA | SMI, HGS, GS | MNA-lf | ≤23.5 | 2.29 (1.03-5.09) | Sex, Age, Smoking, BMI, CC, FFM,  Total body fat, VFA,  Protein, BMC, ALM, SMI, HGS, GS | 14 |
| Saeki | 2023(23) | Japan | Cross-sectional | Cirrhosis | 202 | 69 (11) | 65 | - | Japan Society of Hepatology | BIA | SMI, HGS, GS | GNRI | <102.1 | 0.932 (0.895 - 0.970) | Age, etiology, Child–Pugh score, ALBI score,  BCAA | 5 |
| Senior | 2015(24) | Australia | Cross-sectional | Nursing Home | 102 | 84.5 (8.2) | 70 | - | EWGSOP1 | BIA | ASM/h2, HGS, GS | MNA-sf | ≤7 | 2.91 (0.099-4.7) | SPPB. Handgrip, physical activity, GDS, IPAQ sitting time | 5 |
| Shiroma | 2023(25) | Japan | Cross-sectional | T2D | 479 | 71 (12) | 45 | - | AWGS 2019 | BIA | ASM/h2, HGS, GS | GNRI  CONUT | GNRI<98  CONUT<3 | GNRI: 4.67 (1.98-11.01)  CONUT: 1.81 (0.70-4.68) | Age, sex, diabetes duration, HbA1c, and eGFR | 5 |
| Simsek | 2022(26) | Turkey | Cross-sectional | Nursing Home | 172 | 82.74 (6.85) | 58 | - | EWGSOP2 | BIA | ASM, HGS | MNA-sf | 8-11 | 7.13 (1.36-37.51) | Gender, age, protein intake, total fluid intake | 4 |
| Simsek | 2019(27) | Turkey | Cross-sectional | Community-dwelling | 909 | 72.8 (6.2) | 64 | - | EWGSOP1 | CC | HGS, GS | MNA-sf | ≤11 | 8.006 (3.069-20.885) | Age,  Number of drugs, Number of chronic diseases, educational status, Ownership of the house, BMI, physical activity, malnutrition, hypertension, diabetes mellitus | 10 |
| Smoliner | 2014(28) | Germany | Cross-sectional | Hospitalized | 198 | 82.8 (5.9) | 70 | - | EWGSOP1 | BIA | ASM/h2, HGS, SPPB | MNA-sf | ≤7 | 0.914 (0.763-1.094) | Gender, age, number of drugs, length of stay, BMI, MMSE, BI | 7 |
| Sousa-Santos | 2019(29) | Portugal | Cross-sectional | Community-dwelling | 1500 | 79.6 (25.9) | 58 | - | EWGSOP2 | Mid-arm muscle circumference and CC | HGS, GS | MNA-sf | ≤11 | 1.86 (1.01-3.43) | Sex, age, residential status, regional area, educational  level, marital status, self-perception of health status,  smoking status, alcohol consumption, BMI classification,  physical activity level,  and cognitive function | 12 |
| Su | 2019(30) | Japan | Cross-sectional | Community-dwelling | 310 | 76 (5.8) | 71 | - | EWGSOP2 | BIA | ASM/h2, HGS | MNA-sf | ≤11 | 4.37 (0.77-24.78) | - | 0 |
| Tan | 2021(31) | Singapore | Cross-sectional | Community-dwelling | 541 | 58.6 (18.7) | 58 | - | AWGS 2019 | DXA | SMI, HGS, GS | MNA-sf | ≤11 | 2.31 (1.22-4.37) | Age, gender, physical activity level, cognition and FMI | 5 |
| Tao | 2020(32) | China | Prospective | Hospitalized | 427 | 84.4 (6.1) | 77 | 24.9 months | EWGSOP2 | CC | HGS | NRS-2002 | >2 | 3.23 (1.41-7.38) | Age, CI, COPD,  neoplasms, BMI, WC, Hb, ALB, PAB, Cr, Iron | 11 |
| Torii | 2019(33) | Japan | Cross-sectional | Rheumatoid Arthritis | 388 | 63.7 (17.1) | 100 | - | EWGSOP1 | BIA | SMI, HGS, GS | MNA-sf | ≤11 | 0.61 (0.51-0.71) | Age, duration of rheumatoid arthritis, DAS28-ESR, SJC, TJC, PT-VAS, Dr-VAS, ESR, Stage, HAQ, glucocorticoids, SMI, Right hand grip, Left hand grip, gait speed, BMI, Falls, Fractures | 18 |
| Tramontano | 2017(34) | Perù | Cross-sectional | Community-dwelling | 222 | 75.4 (6.9) | 54 | - | IWGS | BIA | ASM/h2, GS | MNA-lf | ≤23.5 | 3.2 (0.8-12.4) | Age, gender, physical activity level,  BMI, 6MWT, disability in ADL, disability in  IADL, number of children, number of drugs, number of chronic diseases | 10 |
| Velazquez-Alva | 2020(35) | Mexico | Cross-sectional | Nursing Home | 114 | 84.1 (7) | 100 | - | EWGSOP1 | CC | HGS, GS | MNA-lf | ≤23.5 | 4.07 (1.64-10.01) | Age, T2DM | 2 |
| Wu | 2014(36) | Taiwan | Cross-sectional | Community-dwelling | 549 | 76 (6.2) | 48 | - | EWGSOP1 | BIA | ASM/h2, HGS, GS | MNA | MNA score analysed as continuous variable | 1.125 (0.9-1.407) | Age, waist circumference, sex, body mass index, Working regularly, Habitual smoking, Alcohol drinking, hypertension, diabetes, SPMSQ, SPPB | 11 |
| Xiang | 2022(37) | China | Cross-sectional | Community-dwelling | 3829 | 67 (12) | 64 | - | AWGS 2019 | BIA | ASM/h2, HGS or 5CST | GNRI | <92 | 6.43 (2.57–16.09) | Age, sex, ethnicity (compared to Han), marriage status (compared to being married), smoking, drinking alcohol, ADL impairment, IADL  impairment, moderate to severe cognitive impairment, number of comorbidities (compared to no comorbidity), moderate to severe anxiety, moderate to severe  depression, indirect bilirubin, ALT, creatinine, glucose, triglycerides, HDL, WBC, absolute neutrophil count, absolute lymphocyte count, RBC, RDW-CV, platelets, platelet distribution  width, FT3, FT4, INS, plasma total cortisol and Vit D | 32 |
| Xu | 2020(38) | China | Cross-sectional | Community-dwelling | 582 | 86.4 (3.5) | 58 | - | AWGS 2014 | BIA | SMI, HGS, GS | MNA | MNA score analysed as continuous variable | 0.78 (0.64-0.96) | Age, sex  education, marital status, smoking status, drinking  status in the past year, physical activity status, hypertension, CHD, stroke/TIA, COPD, CKD, diabetes, osteoarthritis, tumor  of any type, BMI | 16 |
| Zeng | 2018(39) | China | Cross-sectional | Nursing Home | 277 | 81.6 (3.3) | 70 | - | EWGSOP1 | BIA | ASM/h2, HGS, GS | MNA-lf | ≤17 | 4.02 (1.05-15.39) | Age, smoking, haemoglobin, creatinine, CC, HS, GS, ASM | 8 |
| Cano-Garcia | 2023(40) | Spain | Cross-sectional | Rheumatoid arthritis | 154 | 71 (4.8) | 77.9 | - | EWGSOP2 | DXA | SMI, HGS | MNA-sf | ≤11 | 0.7 (0.5 - 0.9) | Sex, age, RA duration, DAS28-ESR, SPPB | 5 |
| Sri-on | 2022(41) | Thailand | Cross-sectional | Community-dwelling | 892 | 69 (9) | 68 | - | AWGS 2019 | BIA | ASM/h2, HGS | MNA-lf | ≤23.5 | 1.75 (1.24-2.48) | Age, BMI, CCI, Frailty, sensory impairment, sitting time, Berg Balance Test, TUG, proximal muscle weakness | 9 |
| Borges | 2022(42) | Spain | Cross-sectional | Hospitalised w Hip Fracture | 90 | 83.4 (7.2) | 88 | - | EWGSOP2 | BIA | SMI, HGS | MNA-sf | ≤7 | 1.976 (0.323-13.232) | BMI and CC | 2 |
| Lu | 2022(43) | China | Cross-sectional | Hospitalised | 441 | 81.3 (9.5) | 40.5 | - | AWGS 2019 | BIA | ASM/h2, HGS or GS | MNA-sf | ≤7 | 8.52 (0.90 - 81.14) | Age, gender, education, smoking, BMI, ALT, CRP, Hb,  and ALB | 9 |
| Huo | 2015(44) | Australia | Cross-sectional | Community-dwelling | 239 | 80.4 (7.1) | 46 | - | EWGSOP1 | DXA | SMI, HGS, GS | MNA-sf | ≤11 | 1.53 (0.78-3.02) | Age and gender | 2 |
| Lu | 2020(45) | Singapore | Cross-sectional | Community-dwelling | 189 | 73.2 (5.3) | 62.9 | - | AWGS 2014 | DXA | SMI, Lower limp strength, and GS | MNA-sf | ≤11 | 9.877 (3.210–30.390) | Age and gender | 2 |
| Demirdag | 2022(46) | Turkey | Cross-sectional | Community-dwelling | 341 | 74,4 (6.57) | 68.9 | - | EWGSOP2 | BIA | SMI, HGS or GS | MNA | MNA score analysed as continuous variable | 0.835 (0.784-0.89) | - | 0 |
| Calcaterra | 2024(47) | France | Cross-sectional | Community-dwelling patients | 809 | 81.8 (6.9) | 65 | - | EWGSOP2 | DXA | SMI, HGS | MNA-lf | ≤23.5 | 3.68 (2.30-5.89) | Age, sex, marital status, living status, ADL, IADL, SPPB, MMSE, Charlson index | 9 |
| Vidaña-Espinoza | 2024(48) | Mexico | Cross-sectional | Community-dwelling patients | 256 | 76.6 (7.9) | 83.9 | - | EWGSOP2 | DXA | CHGS, ASMI | MNA-lf | ≤23.5 | 1.41 (0.61-3.25) | Age, sex, and waist circumference | 3 |

**Abbreviations**: EWGSOP: European Working Group on Sarcopenia in Older People; AWGS: Asian Working Group for Sarcopenia; SGA: Subjective Global Assessment; NRS-2002: Nutritional Risk Screening 2002; G8: Geriatric-8 questionnaire; GFI: Groningen Frailty Index; MUST: Malnutrition Universal Screening Tool; MNA: Mini Nutritional Assessment; CT: computed tomography; BIA: bioelectrical impedance; DXA: dual-energy X-ray absorptiometry; HGS: handgrip strength; GS: gait speed; SPPB: Short Physical Performance Battery; 5CST: 5-time Chair Stand Test; SMM: skeletal muscle mass; FM: fat mass; BCM: body cell mass; ALM: appendicular lean mass; SMI: skeletal muscle index; ASM: appendicular skeletal mass; ASMI: appendicular skeletal mass index; CC: calf circumference; BMI: body mass index; COPD: chronic obstructive pulmonary disease; CKD: chronic kidney disease; HF: heart failure; CHD: coronary heart disease; CVI: cerebrovascular incident; T2D: type 2 diabetes; CRP: c-reactive protein; Charlson’s Comorbidity Index: CCI; Barthel Index: BI; MMSE: mini-mental state examination; ASA: American Society of Anesthesiologists; WBC: white blood cells; HDL: high density lipoprotein; DAS28-ESR: Disease Activity Score 28 - Erythrocyte Sedimentation Rate; SJC: swollen joint count; TJC: tender joint count; PG-VAS: Patient Global Visual Analogue Scale; HAQ: Health Assessment Questionnaire; HbA1c: glycated hemoglobin; ADL: Activities of Daily Living; IADL: Instrumental Activities of Daily Living.

**Supplementary Table 3.** Studies that examined the prognostic impact of malnutrition and sarcopenia versus sarcopenia alone on all-cause mortality.

| **Author, year** | **Country** | **Study design** | **Source of Cohort** | **Sample Size** | **Mean Age (SD)** | **Females %** | **Follow-up Measurement (months)** | **Sarcopenia Definition** | **Muscle Mass Assessment Tool** | **Sarcopenia Indices** | **Malnutrition Tool** | **Malnutrition Definition** | **Odds ratio of Sarcopenia with Malnutrition (95% CI)** | **Type of Adjustments** | **Hazard Ratio of Sarcopenia without malnutrition (95% CI)/Prevalence of malnutrition in sarcopenia** | **Type of Adjustments** |
| --- | --- | --- | --- | --- | --- | --- | --- | --- | --- | --- | --- | --- | --- | --- | --- | --- |
| Gümüssoy, 2021(49) | Turkey | Prospective | Hospitalized | 350 | 77.2  (7.6) | 56 | 24 | EWGSOP2 | BIA | ALM, HGS, GS | MNA-sf | ≤7 | 19.9 (7.5-52.7) | Age, gender, hypertension, diabetes, HF, dementia, COPD, CKD, CVI, albumin, and CRP | 13.4 (5.50-32.80) | Age, gender, hypertension, diabetes, HF, dementia, COPD, CKD, CVI, albumin, and CRP |
| Hu, 2017(50) | China | Prospective | Hospitalized | 453 | 79 (7.8) | 30 | 36 | AWGS 2014 | Anthropometric equation | ASMI, HGS, GS | MNA-lf | <23.5 | 4.25 (2.22-8.12) | Age, gender, education level, smoking status, alcohol drinking status, physical activity status,  comorbidities, ADL scores, IADL scores, GDS-30 scores, and MMSE scores | 1.66 (0.48-5.72) | Age, gender, education level, smoking status, alcohol drinking status, physical activity status,  comorbidities, ADL scores, IADL scores, GDS-30 scores, and MMSE scores |
| Macedo, 2021(51) | Brazil | Longitudinal | Hemodialysis | 170 | 70.6 (7.2) | 34.7 | 23.5 | EWGSOP2 | BIA | ASMI, HGS | 7p-SGA | ≤5 | 2.99 (1.23 - 7.25) | Gender, age, and hs-CRP | 2.65 (0.86- 7.05) | Gender, age, and hs-CRP |
| Sobestiansky, 2021(52) | Sweden | Prospective | Rehabilitation | 56 | 84  (7.30) | 68 | 24 | EWGSOP2 | DXA | HGS, 5CST, ASMI/CC | MNA-sf | ≤7 | 4.83 (1.04-22.39) | Age and CCI score | 2.13 (0.71-6.37) | Age and CCI score |
| Sousa, 2022(53) | Brazil | Longitudinal | Hospitalized | 550 | 55.3 ± 14.9 | 47 | 6 | EWGSOP2 | CC | HGS, TUG | SGA | >3 | 1.15 (1.08–1.21) | CCI and surgical  procedure | 56.7% of sarcopenic had malnutrition (SGA B or C) vs. 29.4% in non-sarcopenic | - |

**Abbreviations**: EWGSOP: European Working Group on Sarcopenia in Older People; AWGS: Asian Working Group for Sarcopenia; SGA: subjective global assessment; MNA-LF: Mini Nutritional Assessment - Long Form; MNA-SF: Mini Nutritional Assessment - Short Form; 7p-SGA: The 7-point scale SGA form; CT: computed tomography; BIA: bioelectrical impedance; DXA: dual-energy X-ray absorptiometry; HGS: handgrip strength; GS: gait speed; SPPB: Short Physical Performance Battery; SMM: skeletal muscle mass; FM: fat mass; BCM: body cell mass; ALM: appendicular lean mass; SMI: skeletal muscle index; ASM: appendicular skeletal mass; ASMI: appendicular skeletal mass index; CC: calf circumference; BMI: body mass index; COPD: chronic obstructive pulmonary disease; CKD: chronic kidney disease; HF: heart failure; CHD: coronary heart disease; CVI: cerebrovascular incident; T2D: type 2 diabetes; (hs)CRP: (high sensitivity) c-reactive protein; Charlson’s Comorbidity Index: CCI; Barthel Index: BI; MMSE: mini-mental state examination; ASA: American Society of Anesthesiologists; ADL: Activities of Daily Living; IADL: Instrumental Activities of Daily Living.

**Supplementary Table 4.** The effect of malnutrition in increasing the odds of sarcopenia (continuous independent variables).

|  | **Number of studies (cohorts)** | **Odds ratio**  **(95% CI)** | **p-value** | **I^2^** | **Egger’s test (SE)**  **(p-value)** | **Re-calculated OR**  **(trimmed)** |
| --- | --- | --- | --- | --- | --- | --- |
| **Malnutrition** | 12 (12) | 1.38  (1.18-1.61) | <0.01 | 94.8 | 2.94  (1.66)  (0.10) | Unchanged |

**Abbreviations:** CI=confidence interval; SE=standard error; OR=odds ratio; p=p-value; I**^2^**: heterogeneity.

|  | **Mean age** | **Percentage of females** | **Sample Size** | **Number of Adjustments** | **Setting** | **Malnutrition tool** | **Sarcopenia definition** |
| --- | --- | --- | --- | --- | --- | --- | --- |
| **Malnutrition** | -0.0091534 ± 0.0140568  (p=0.53)  R^2^=0.00 | 0.0012811 ± 0.0053081  (p=0.81)  R^2^=0.00 | 0.0005039 ± 0.0002137  (p= 0.04)  R^2^=50.86 | -0.0020244 ± 0.0177821  (p= 0.91)  R^2^=0.00 | 0.002 ± 0.007  (p=0.91)  R^2^=0.00 | 0.10 ± 0.78  (p=0.81)  R^2^=0.00 | -0.02 ± 0.07  (p=0.83)  R^2^=0.00 |

**Supplementary Table 5.** Meta-regression analysis of potential moderators of malnutrition effect in increasing the odds of sarcopenia (continuous independent variables).

|  | **Number of studies (cohorts)** | **Odds ratio**  **(95% CI)** | **p-value** | **I^2^** | **Egger’s test (SE)**  **(p-value)** | **Re-calculated OR**  **(trimmed)** |
| --- | --- | --- | --- | --- | --- | --- |
| **Malnutrition** | 37 (38) | 2.99  (2.26-3.96) | <0.001 | 78.3 | 2.78  (0.51)  (<0.01) | OR=2.23  (1.74- 2.88)  (12L) |

**Supplementary Table 6.** The effect of malnutrition in increasing the odds of sarcopenia (categorical independent variables).

**Abbreviations:** CI=confidence interval; SE=standard error; OR=odds ratio; p=p-value; I**^2^**: heterogeneity.

**Supplementary Table 7.** Meta-regression analysis of potential moderators of malnutrition in increasing the odds of sarcopenia (categorical independent variables).

|  | **Mean age** | **Percentage of females** | **Sample Size** | **Number of Adjustments** | **Setting** | **Malnutrition tool** | **Sarcopenia definition** |
| --- | --- | --- | --- | --- | --- | --- | --- |
| **Malnutrition** | -0.0230186 ± 0.0156662 (p=0.15)  R^2^=8.86 | 0.0045361 ± 0.0072267  (p=0.53)  R^2^=0.00 | 0.000219 ± 0.0001801  (p= 0.23)  R^2^=3.56 | 0.026923 ± 0.0190127  (p= 0.17)  R^2^=6.63 | -0.007 ± 0.010  (p=0.27)  R^2^=0.00 | 0.26 ± 0.87  (p=0.78)  R^2^=0.00 | -0.21 ± 0.74  (p=0.45)  R^2^=0.00 |

**Abbreviations:** R^2^=R-squared (adjusted); p=p value

|  | **Number of studies** | **Hazard Ratio**  **(95% CI)** | **p-value** | **I^2^** | **Egger’s test (SE)**  **(p-value)** | **Re-calculated HR**  **(trimmed)** |
| --- | --- | --- | --- | --- | --- | --- |
| **Sarcopenia and Malnutrition Combined** | 5 | 4.04  (1.36-11.94) | <0.01 | 92.8 | 3.23  (0.88)  (0.02) | Unchanged |

**Supplementary Table 8.** Effect of sarcopenia and malnutrition combined on all-cause mortality.

**Abbreviations:** CI=confidence interval; SE=standard error; HR=Hazard ratio; p=p-value; I**^2^**: heterogeneity.

|  | **Mean age** | **Percentage of females** | **Follow-up months** | **Number of adjustments** | **Setting** | **Malnutrition tool** | **Sarcopenia definition** |
| --- | --- | --- | --- | --- | --- | --- | --- |
| **Sarcopenia and Malnutrition Combined** | 0.0648713 ±0.0359214  (p=0.15)  R^2^=38.87 | 0.0177743 ± 0.0374398  (p=0.66)  R^2^=0.00 | 0.0572674 ± 0.0337728  (p= 0.17)  R^2^=34.87 | 0.1959356 ± 0.0727567 (p=0.05)  R^2^=71.01 | 0.25± 0.34  (p=0.65)  R^2^=0.00 | 0.89 ± 1.03  (p=0.22)  R^2^=0.00 | 0.87 ± 1.25  (p=0.83)  R^2^=0.00 |

**Supplementary Table 9.** Meta-regression analysis of potential moderators of sarcopenia and malnutrition combined on all-cause mortality.

**Abbreviations:** R^2^=R-squared (adjusted); p=p value

**Supplementary Figure 1.** Funnel plot using malnutrition as a categorical variable.


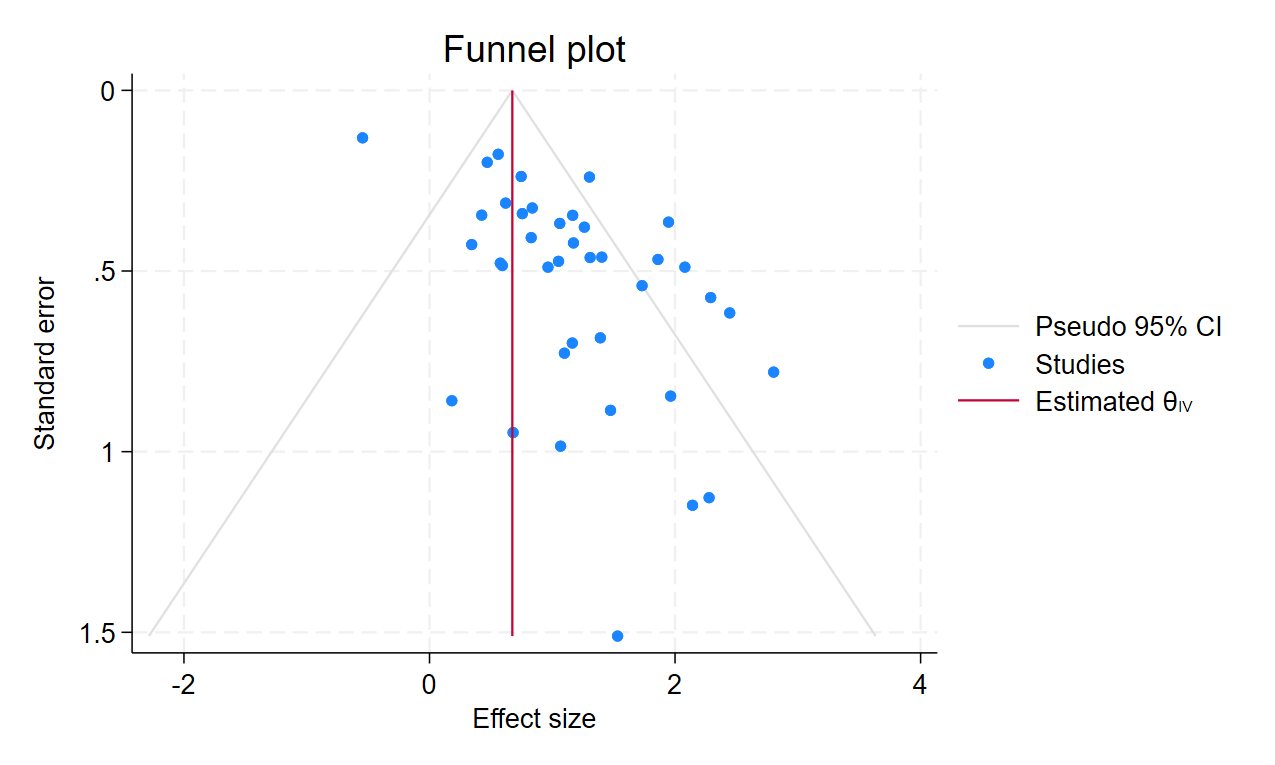


**Supplementary Figure 2.** Funnel plot using malnutrition as a continuous variable.

**
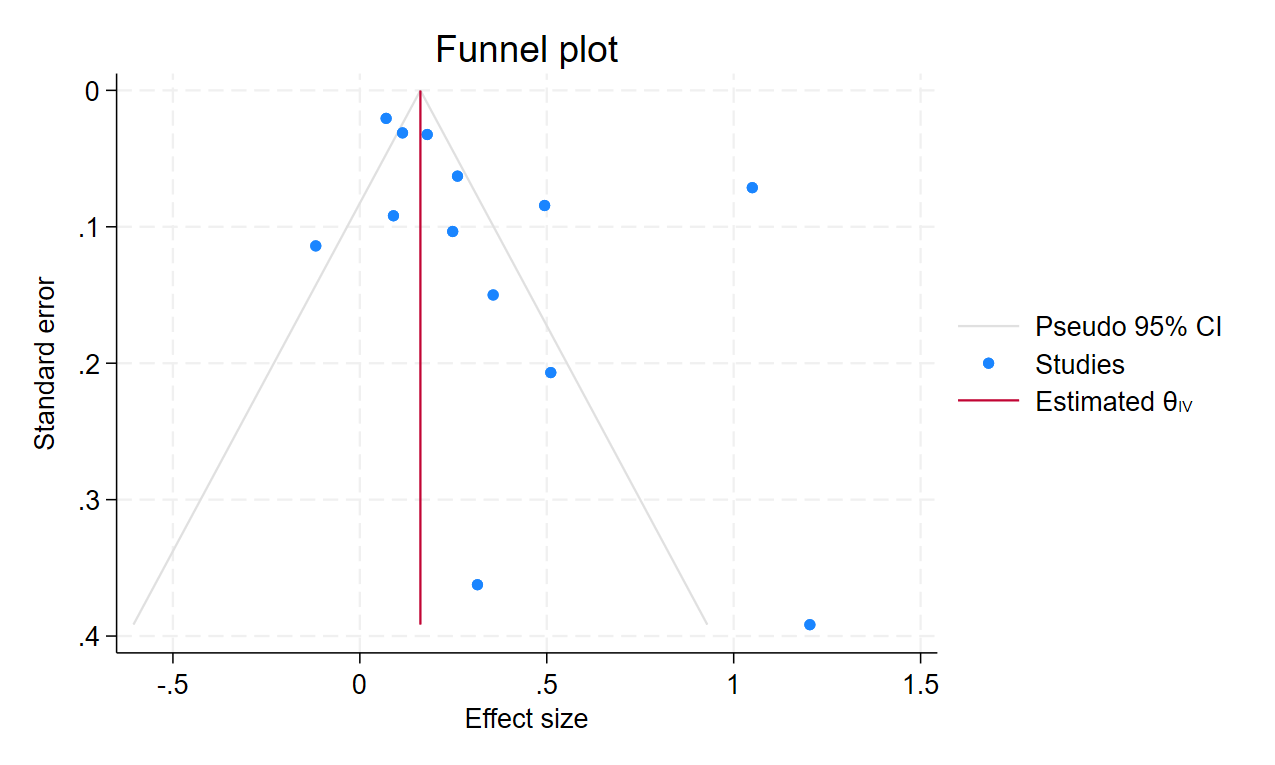
**

**Supplementary Figure 3.** Funnel plot using malnutrition as a categorical variable, following trim-and-fill.

**
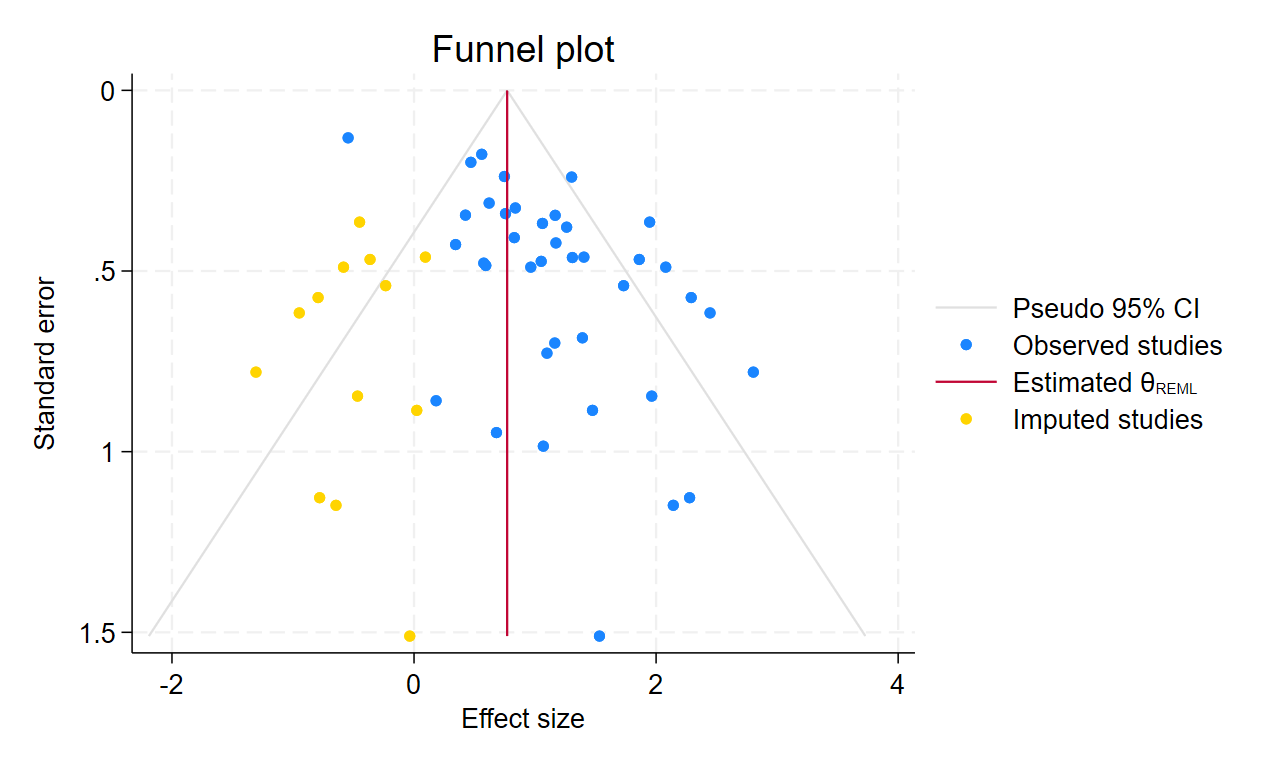
**

**Supplementary Figure 4.** Funnel plot using malnutrition as a continuous variable, following trim-and-fill.


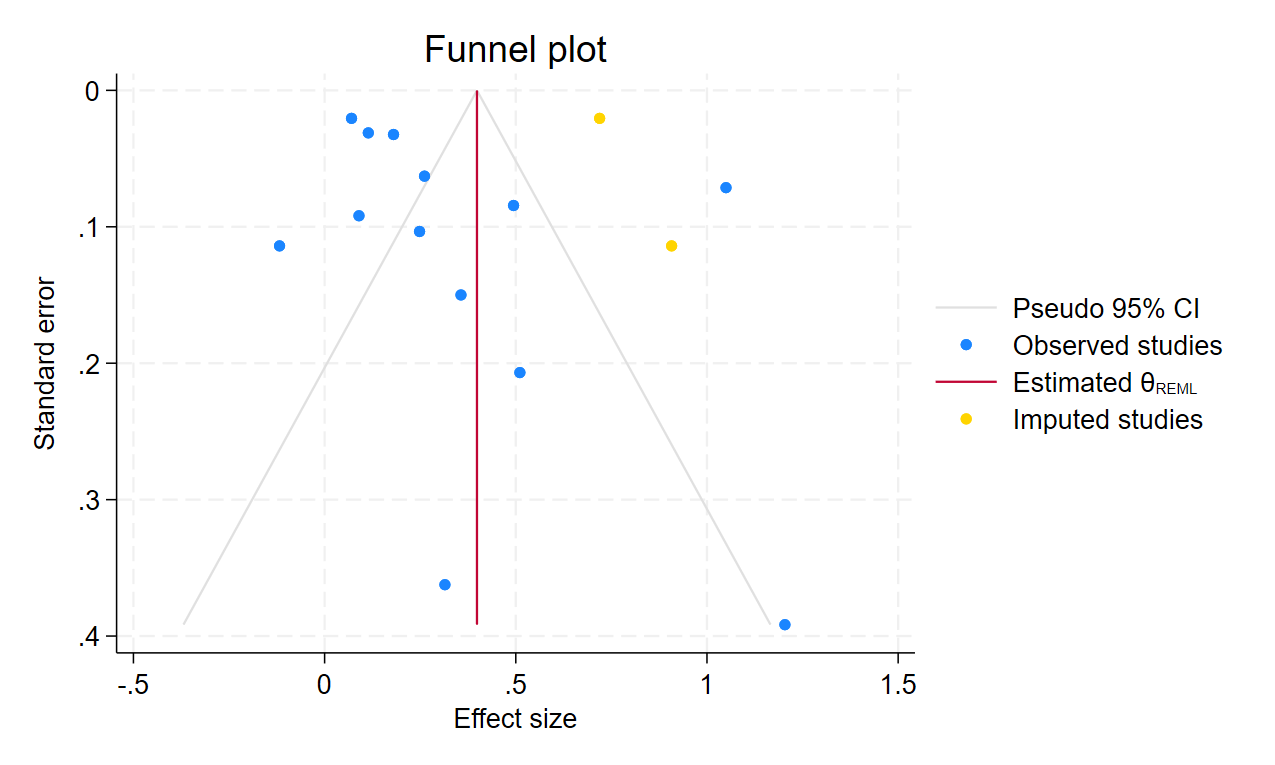


**Supplementary Table 10.** Risk of bias of prospective studies utilizing original version of the Newcastle Ottawa Scale.

| **First**  **Author** | **Representativeness of the exposed cohort** | **Selection of the non-exposed cohort** | **Ascertainment of exposure** | **Demonstration that outcome of interest was not present at the start of the study** | **Comparability of cohorts based on the design or analysis** | **Assessment of outcome** | **Was follow-up long enough for outcomes to occur** | **Adequacy of follow-up of cohorts** | **Total score** |
| --- | --- | --- | --- | --- | --- | --- | --- | --- | --- |
| Chaiwat, 2021 | * | * | - | * | * | - | * | - | 5 |
| Gümüssoy, 2021 | * | * | * | * | ** | * | * | - | 8 |
| Hu, 2017 | * | * | - | * | - | * | * | * | 6 |
| Malafarina, 2019 | * | * | * | * | ** | - | * | * | 9 |
| Sobestiansky, 2021 | - | - | * | * | ** | - | * | * | 6 |
| De Araujo, 2021 | * | * | - | * | ** | - | * | * | 7 |
| Lee, 2023 | - | * | * | * | ** | * | * | * | 7 |
| Macedo, 2021 | * | * | * | * | ** | * | * | - | 8 |
| Sousa, 2022 | * | * | * | * | ** | * | * | * | 9 |
| Tao, 2020 | * | * | * | * | ** | * | * | - | 8 |
| Vidana-Espinoza, 2024 | * | * | * | * | ** | * | * | _ | 8 |

**Supplementary Table 11.** Risk of bias utilizing a modified version of the Newcastle Ottawa Scale for cross-sectional studies*

| **First**  **Author** | **Representativeness of the sample** | **Sample size** | **Non-responders** | **Ascertainment of the exposure** | **Comparability based on the study design or analysis** | **Assessment of outcome** | **Statistical test** | **Total score** |
| --- | --- | --- | --- | --- | --- | --- | --- | --- |
| Alexandre, 2014 | * | * | * | ** | ** | - | * | 8 |
| Cao, 2022 | * | * | * | ** | ** | - | * | 8 |
| Chang, 2021 | * | - | * | ** | ** | ** | * | 9 |
| Chen, 2023 | * | - | * | ** | * | - | * | 6 |
| Darroch, 2022 | * | - | * | ** | * | ** | * | 8 |
| Escribà-Salvans, 2022 | * | * | * | ** | * | ** | * | 9 |
| Gao, 2015 | * | - | * | ** | * | - | * | 6 |
| Isleyen, 2023 | * | - | * | ** | * | - | * | 6 |
| Kamo, 2018 | * | - | * | ** | * | - | * | 6 |
| Kirk. 2020 | * | - | * | - | * | * | * | 5 |
| Kuo, 2018 | * | - | * | ** | * | ** | * | 8 |
| Lardies-Sanchez, 2017 | * | * | * | ** | * | ** | * | 9 |
| Nasimi, 2019 | * | * | * | ** | * | ** | * | 9 |
| Senior, 2015 | * | * | * | ** | - | ** | * | 8 |
| Simsek, 2022 | * | * | * | ** | * | ** | * | 9 |
| Simsek, 2019 | * | * | * | ** | * | - | * | 7 |
| Smoliner, 2014 | * | - | * | ** | - | ** | * | 7 |
| Sousa-Santos, 2019 | * | - | * | ** | * | - | * | 6 |
| Su, 2019 | * | * | * | ** | * | ** | * | 8 |
| Tan, 2021 | * | * | * | ** | * | ** | * | 9 |
| Torii, 2019 | * | - | * | ** | * | ** | * | 8 |
| Tramontano, 2017 | * | - | * | ** | * | ** | * | 8 |
| Velazquez-Alva, 2020 | * | * | * | ** | * | - | * | 7 |
| Wu, 2014 | * | - | * | ** | * | ** | * | 8 |
| Xu, 2020 | * | - | * | ** | - | ** | * | 7 |
| Zeng, 2018 | * | - | * | ** | * | ** | * | 8 |
| Bellanti, 2020 | * | - | - | ** | - | - | * | 4 |
| Borges, 2022 | * | - | - | ** | * | - | * | 5 |
| Calcaterra, 2024 | * | * | - | ** | * | - | * | 6 |
| Cano-García, 2023 | * | - | - | ** | * | - | * | 5 |
| Chew, 2022 | * | * | - | ** | * | - | * | 6 |
| Demirdag, 2022 | * | * | - | ** | * | - | * | 6 |
| Huo, 2015 | * | * | - | ** | * | - | * | 6 |
| Kim, 2014 | - | - | - | ** | * | - | * | 4 |
| Lu, 2022 | * | * | - | ** | * | - | * | 6 |
| Matsuura, 2022 | - | - | - | ** | * | - | * | 4 |
| Miwa, 2022 | * | * | - | * | * | - | * | 5 |
| Saeki, 2023 | * | - | - | ** | * | - | * | 5 |
| Shiroma, 2023 | * | * | - | ** | * | - | * | 6 |
| Sri-on, 2022 | * | * | - | ** | * | - | * | 6 |
| Xiang, 2022 | * | * | - | ** | * | - | * | 6 |

*References:
Modesti PA, Reboldi G, Cappuccio FP, Agyemang C, Remuzzi G, Rapi S, et al. Panethnic differences in blood pressure in Europe: a systematic review and meta-analysis. PLoS One 2016;11:e0147601. 
Herzog R, Alvarez-Pasquin MJ, D ıaz C, Del Barrio JL, Estrada JM, Gil A. Are healthcare workers’ intentions to vaccinate related to their knowledge, beliefs and attitudes? A systematic review. BMC Public Health 2013;13:154.

**Supplementary File 1**

**Excluded studies**

Reasons for exclusion

Used relative risk: (1)

Used hazard ratio instead of odds ratio to answer the “odds of sarcopenia attributable to malnutrition”: (2, 3)

Identical cohort with an included study: (4-6)

Used probable sarcopenia: (7, 8)

Population age was 18-90 years: (9)

Used SARC-F to define sarcopenia: (10-12)

Incomplete definition of sarcopenia: (13, 14)

L3 psoas muscle index was used to define sarcopenia: (15-17)

Used handgrip strength to define sarcopenia: (18)

Limited information about what type of malnutrition tool was used: (19)

Analysed data based on individuals with pre-sarcopenia and sarcopenia combined: (20, 21)

Calculated the hazard ratio of sarcopenia and malnutrition combined without sarcopenia alone as a reference: (22)

**References of excluded studies**

1. Tay L, Leung B, Wee S, Tay K, Ali N, Chan M et al. Association of nutrition and immune-endocrine dysfunction with muscle mass and performance in cognitively impaired older adults. Archives of gerontology and geriatrics. 2018;75:20-7.

2. Beaudart C, Sanchez-Rodriguez D, Locquet M, Reginster J-Y, Lengelé L, Bruyère O. Malnutrition as a strong predictor of the onset of sarcopenia. Nutrients. 2019;11(12):2883.

3. Lengelé L, Bruyère O, Beaudart C, Reginster J-Y, Locquet M. Malnutrition, assessed by the Global Leadership Initiative on Malnutrition (GLIM) criteria but not by the mini nutritional assessment (MNA), predicts the incidence of sarcopenia over a 5-year period in the SarcoPhAge cohort. Aging clinical and experimental research. 2021;33(6):1507-17.

4. Ogawa A, Shimizu K, Nakagami T, Maruoka H, Shirai K. Physical function and cardio-ankle vascular index in elderly heart failure patients. International Heart Journal. 2020;61(4):769-75.

5. Chen W, Shi S, Tu J, Liao L, Liao Y, Chen K et al. Nutrition-related diseases and cardiovascular mortality in American society: National health and nutrition examination study, 1999–2006. BMC Public Health. 2022;22(1):1849.

6. Li Y, Xiang Q, Dong B, Liang R, Song Q, Deng L et al. Transitional dynamics of sarcopenia and associations of nutritional indices with state transitions in Chinese aged≥ 50. The Journal of nutrition, health and aging. 2023;27(9):741-51.

7. Cheng L, Sit JW, Chan HY, Choi KC, Cheung RK, Wong MM et al. Sarcopenia risk and associated factors among Chinese community-dwelling older adults living alone. Scientific Reports. 2021;11(1):22219.

8. Wong HJ, Harith S, Lua PL, Ibrahim KA. Possible sarcopenia and its association with nutritional status, dietary intakes, physical activity and health-related quality of life among older stroke survivors. Annals of geriatric medicine and research. 2022;26(2):162.

9. Sousa AS, Guerra RS, Fonseca I, Pichel F, Amaral TF. Sarcopenia among hospitalized patients–a cross-sectional study. Clinical nutrition. 2015;34(6):1239-44.

10. Sahin UK, Tozluoglu EY, Durdu H, Korkmaz N, Bahar NT, Yavuz E. Screening for frailty and sarcopenia in community-dwelling older adults: a cross-sectional study from the Eastern Black Sea region of Turkey. Aging clinical and experimental research. 2022;34(9):2047-56.

11. Ozer FF, Akin S, Tasci İ, Tasar PT, Savas S, Cincin AT et al. Risk of sarcopenia in hospitalized patients and related clinical factors: a multicenter study from Turkey. European Geriatric Medicine. 2021;12:863-70.

12. Sato PHR, Ferreira AA, Rosado EL. The prevalence and risk factors for sarcopenia in older adults and long-living older adults. Archives of gerontology and geriatrics. 2020;89:104089.

13. Perna S, Peroni G, Faliva MA, Bartolo A, Naso M, Miccono A et al. Sarcopenia and sarcopenic obesity in comparison: prevalence, metabolic profile, and key differences. A cross-sectional study in Italian hospitalized elderly. Aging clinical and experimental research. 2017;29:1249-58.

14. Zwart AT, van der Hoorn A, van Ooijen PM, Steenbakkers RJ, de Bock GH, Halmos GB. CT‐measured skeletal muscle mass used to assess frailty in patients with head and neck cancer. Journal of cachexia, sarcopenia and muscle. 2019;10(5):1060-9.

15. Wang H, Wang S, Li C, Yang W, Guo G, Hui Y et al. Coexistent GLIM-defined malnutrition and sarcopenia increase the long-term mortality risk in hospitalized patients with decompensated cirrhosis. Annals of Nutrition and Metabolism. 2023;79(5):423-33.

16. Peng H, Tan X. The prognostic significance of sarcopenia and the neutrophil-to-lymphocyte ratio in elderly patients with esophageal squamous cell carcinoma. Cancer Management and Research. 2021:3209-18.

17. Gao B, Chen W, Liu Y, Li Y, Li X, Ding C et al. Associations between nutrition risk scores and sarcopenia in gastrointestinal cancer patients: a cross-sectional study. Supportive Care in Cancer. 2022;30(4):3269-77.

18. Chou Y-Y, Lin C-F, Lee Y-S, Weng SC, Kuo F-H, Hsu C-Y et al. The associations of osteoporosis and possible sarcopenia with disability, nutrition, and cognition in community-dwelling older adults. BMC geriatrics. 2023;23(1):730.

19. Kurose S, Nishikawa S, Nagaoka T, Kusaka M, Kawamura J, Nishioka Y et al. Prevalence and risk factors of sarcopenia in community-dwelling older adults visiting regional medical institutions from the Kadoma Sarcopenia Study. Scientific Reports. 2020;10(1):19129.

20. Yap SF, Boo NY, Pramod DS, Thaw Z, Liew SF, Woo LF et al. Risk factors associated with sarcopenia among independently mobile, institutionalised older people in the Klang valley of Malaysia: a cross-sectional study. The Malaysian Journal of Medical Sciences: MJMS. 2020;27(2):120.

21. Lee H, Kim K, Ahn J, Lee DR, Lee JH, Hwang SD. Association of nutritional status with osteoporosis, sarcopenia, and cognitive impairment in patients on hemodialysis. Asia Pacific Journal of Clinical Nutrition. 2020;29(4):712-23.

22. Beretta MV, de Paula TP, da Costa Rodrigues T, Steemburgo T. Prolonged hospitalization and 1-year mortality are associated with sarcopenia and malnutrition in older patients with type 2 diabetes: A prospective cohort study. Diabetes Research and Clinical Practice. 2024;207:111063.

**References**

1. da Silva Alexandre T, de Oliveira Duarte Y, Ferreira Santos J, Wong R, Lebrão M. Prevalence and associated factors of sarcopenia among elderly in Brazil: findings from the SABE study. *The journal of nutrition, health & aging*. 2014;18:284-90.

2. Bellanti F, Lo Buglio A, Quiete S, Pellegrino G, Dobrakowski M, Kasperczyk A et al. Comparison of three nutritional screening tools with the new glim criteria for malnutrition and association with sarcopenia in hospitalized older patients. *Journal of clinical medicine*. 2020;9(6):1898.

3. Cao W, Zhu A, Chu S, Zhou Q, Zhou Y, Qu X et al. Correlation between nutrition, oral health, and different sarcopenia groups among elderly outpatients of community hospitals: a cross-sectional study of 1505 participants in China. *BMC geriatrics*. 2022;22(1):332.

4. Chaiwat O, Wongyingsinn M, Muangpaisan W, Chalermsri C, Siriussawakul A, Pramyothin P et al. A simpler screening tool for sarcopenia in surgical patients. *Plos one*. 2021;16(9):e0257672.

5. Chang C-F, Yeh Y-L, Chang H-Y, Tsai S-H, Wang J-Y. Prevalence and risk factors of sarcopenia among older adults aged≥ 65 years admitted to daycare centers of Taiwan: Using AWGS 2019 guidelines. *International journal of environmental research and public health*. 2021;18(16):8299.

6. Chen Z, Laurentius T, Fait Y, Müller A, Mückter E, Hao D et al. Sex-specific associations between serum IL-16 levels and sarcopenia in older adults. *Nutrients*. 2023;15(16):3529.

7. Chew STH, Tey SL, Yalawar M, Liu Z, Baggs G, How CH et al. Prevalence and associated factors of sarcopenia in community-dwelling older adults at risk of malnutrition. *BMC geriatrics*. 2022;22(1):997.

8. Darroch P, O’Brien WJ, Mazahery H, Wham C. Sarcopenia prevalence and risk factors among residents in aged care. *Nutrients*. 2022;14(9):1837.

9. de Araújo BE, Teixeira PP, Valduga K, da Silva Fink J, Silva FM. Prevalence, associated factors, and prognostic value of sarcopenia in patients with acute exacerbated chronic obstructive pulmonary disease: A cohort study. *Clinical Nutrition ESPEN*. 2021;42:188-94.

10. Escribà-Salvans A, Jerez-Roig J, Molas-Tuneu M, Farrés-Godayol P, Moreno-Martin P, Goutan-Roura E et al. Sarcopenia and associated factors according to the EWGSOP2 criteria in older people living in nursing homes: a cross-sectional study. *BMC geriatrics*. 2022;22(1):350.

11. Gao L, Jiang J, Yang M, Hao Q, Luo L, Dong B. Prevalence of sarcopenia and associated factors in Chinese community-dwelling elderly: comparison between rural and urban areas. *Journal of the American Medical Directors Association*. 2015;16(11):1003. e1-. e6.

12. Sucuoglu Isleyen Z, Besiroglu M, Yasin AI, Simsek M, Topcu A, Smith L et al. The risk of malnutrition and its clinical implications in older patients with cancer. *Aging Clinical and Experimental Research*. 2023;35(11):2675-83.

13. Kamo T, Ishii H, Suzuki K, Nishida Y. Prevalence of sarcopenia and its association with activities of daily living among japanese nursing home residents. *Geriatric Nursing*. 2018;39(5):528-33.

14. Kim J-K, Choi SR, Choi MJ, Kim SG, Lee YK, Noh JW et al. Prevalence of and factors associated with sarcopenia in elderly patients with end-stage renal disease. *Clinical nutrition*. 2014;33(1):64-8.

15. Kirk B, Zanker J, Hassan EB, Bird S, Brennan-Olsen S, Duque G. Sarcopenia Definitions and Outcomes Consortium (SDOC) criteria are strongly associated with malnutrition, depression, falls, and fractures in high-risk older persons. *Journal of the American Medical Directors Association*. 2021;22(4):741-5.

16. Kuo Y-H, Wang T-F, Liu L-K, Lee W-J, Peng L-N, Chen L-K. Epidemiology of sarcopenia and factors associated with it among community-dwelling older adults in Taiwan. *The American journal of the medical sciences*. 2019;357(2):124-33.

17. Lardiés-Sánchez B, Sanz-París A, Pérez-Nogueras J, Serrano-Oliver A, Torres-Anoro ME, Cruz-Jentoft AJ. Influence of nutritional status in the diagnosis of sarcopenia in nursing home residents. *Nutrition*. 2017;41:51-7.

18. Lee P-C, Lee K-C, Yang T-C, Lu H-S, Cheng T-Y, Chen Y-J et al. Sarcopenia-related gut microbial changes are associated with the risk of complications in people with cirrhosis. *JHEP Reports*. 2023;5(1):100619.

19. Malafarina V, Malafarina C, Biain Ugarte A, Martinez JA, Abete Goñi I, Zulet MA. Factors associated with sarcopenia and 7-year mortality in very old patients with hip fracture admitted to rehabilitation units: A pragmatic study. *Nutrients*. 2019;11(9):2243.

20. Matsuura S, Shibazaki K, Uchida R, Imai Y, Mukoyama T, Shibata S et al. Sarcopenia is associated with the Geriatric Nutritional Risk Index in elderly patients with poorly controlled type 2 diabetes mellitus. *Journal of Diabetes Investigation*. 2022;13(8):1366-73.

21. Miwa T, Hanai T, Nishimura K, Unome S, Maeda T, Ogiso Y et al. Usefulness of the Global Leadership Initiative on Malnutrition criteria to predict sarcopenia and mortality in patients with chronic liver disease. *Hepatology Research*. 2022;52(11):928-36.

22. Nasimi N, Dabbaghmanesh MH, Sohrabi Z. Nutritional status and body fat mass: Determinants of sarcopenia in community-dwelling older adults. *Experimental gerontology*. 2019;122:67-73.

23. Saeki C, Kinoshita A, Kanai T, Ueda K, Nakano M, Oikawa T et al. The geriatric nutritional risk index predicts sarcopenia in patients with cirrhosis. *Scientific Reports*. 2023;13(1):3888.

24. Senior HE, Henwood TR, Beller EM, Mitchell GK, Keogh JW. Prevalence and risk factors of sarcopenia among adults living in nursing homes. *Maturitas*. 2015;82(4):418-23.

25. Shiroma K, Tanabe H, Takiguchi Y, Yamaguchi M, Sato M, Saito H et al. A nutritional assessment tool, GNRI, predicts sarcopenia and its components in type 2 diabetes mellitus: A Japanese cross-sectional study. *Frontiers in Nutrition*. 2023;10:1087471.

26. Şimşek H, Uçar A. Nutritional status and quality of life are associated with risk of sarcopenia in nursing home residents: a cross-sectional study. *Nutrition Research*. 2022;101:14-22.

27. Simsek H, Meseri R, Sahin S, Kilavuz A, Bicakli DH, Uyar M et al. Prevalence of sarcopenia and related factors in community-dwelling elderly individuals. *Saudi medical journal*. 2019;40(6):568.

28. Smoliner C, Sieber CC, Wirth R. Prevalence of sarcopenia in geriatric hospitalized patients. *Journal of the American Medical Directors Association*. 2014;15(4):267-72.

29. Sousa‐Santos AR, Afonso C, Borges N, Santos A, Padrão P, Moreira P et al. Factors associated with sarcopenia and undernutrition in older adults. *Nutrition & Dietetics*. 2019;76(5):604-12.

30. Su Y, Hirayama K, Han T-f, Izutsu M, Yuki M. Sarcopenia prevalence and risk factors among Japanese community dwelling older adults living in a snow-covered city according to EWGSOP2. *Journal of clinical medicine*. 2019;8(3):291.

31. Tan V, Pang B, Lau L, Jabbar K, Seah W, Chen K et al. Malnutrition and sarcopenia in community-dwelling adults in Singapore: Yishun health study. *The Journal of nutrition, health and aging*. 2021;25(3):374-81.

32. Tao J, Ke Y-Y, Zhang Z, Zhang Y, Wang Y-Y, Ren C-X et al. Comparison of the value of malnutrition and sarcopenia for predicting mortality in hospitalized old adults over 80 years. *Experimental Gerontology*. 2020;138:111007.

33. Torii M, Hashimoto M, Hanai A, Fujii T, Furu M, Ito H et al. Prevalence and factors associated with sarcopenia in patients with rheumatoid arthritis. *Modern Rheumatology*. 2019;29(4):589-95.

34. Tramontano A, Veronese N, Sergi G, Manzato E, Rodriguez-Hurtado D, Maggi S et al. Prevalence of sarcopenia and associated factors in the healthy older adults of the Peruvian Andes. *Archives of gerontology and geriatrics*. 2017;68:49-54.

35. Velázquez‐Alva MC, Irigoyen‐Camacho ME, Zepeda‐Zepeda MA, Lazarevich I, Arrieta‐Cruz I, D'Hyver C. Sarcopenia, nutritional status and type 2 diabetes mellitus: A cross‐sectional study in a group of Mexican women residing in a nursing home. *Nutrition & dietetics*. 2020;77(5):515-22.

36. Wu CH, Chen KT, Hou MT, Chang YF, Chang CS, Liu PY et al. Prevalence and associated factors of sarcopenia and severe sarcopenia in older T aiwanese living in rural community: The T ianliao O ld P eople study 04. *Geriatrics & gerontology international*. 2014;14:69-75.

37. Xiang Q, Li Y, Xia X, Deng C, Wu X, Hou L et al. Associations of geriatric nutrition risk index and other nutritional risk-related indexes with sarcopenia presence and their value in sarcopenia diagnosis. *BMC geriatrics*. 2022;22(1):327.

38. Xu W, Chen T, Cai Y, Hu Y, Fan L, Wu C. Sarcopenia in community-dwelling oldest old is associated with disability and poor physical function. *The Journal of nutrition, health and aging*. 2020;24(3):339-45.

39. Zeng Y, Hu X, Xie L, Han Z, Zuo Y, Yang M. The prevalence of sarcopenia in Chinese elderly nursing home residents: a comparison of 4 diagnostic criteria. *Journal of the American Medical Directors Association*. 2018;19(8):690-5.

40. Cano-García L, Manrique-Arija S, Domínguez-Quesada C, Vacas-Pérez JC, Armenteros-Ortiz PJ, Ruiz-Vilchez D et al. Sarcopenia and nutrition in elderly rheumatoid arthritis patients: a cross-sectional study to determine prevalence and risk factors. *Nutrients*. 2023;15(11):2440.

41. Sri-On J, Fusakul Y, Kredarunsooksree T, Paksopis T, Ruangsiri R. The prevalence and risk factors of sarcopenia among Thai community-dwelling older adults as defined by the Asian Working Group for Sarcopenia (AWGS-2019) criteria: A cross-sectional study. *BMC geriatrics*. 2022;22(1):786.

42. Borges K, Artacho R, Jodar-Graus R, Molina-Montes E, Ruiz-López MD. Calf circumference, a valuable tool to predict sarcopenia in older people hospitalized with hip fracture. *Nutrients*. 2022;14(20):4255.

43. Lu B, Shen L, Zhu H, Xi L, Wang W, Ouyang X. Association between serum homocysteine and sarcopenia among hospitalized older Chinese adults: a cross-sectional study. *BMC geriatrics*. 2022;22(1):896.

44. Huo YR, Suriyaarachchi P, Gomez F, Curcio CL, Boersma D, Gunawardene P et al. Comprehensive nutritional status in sarco-osteoporotic older fallers. *The Journal of nutrition, health and aging*. 2015;19(4):474-80.

45. Lu Y, Karagounis LG, Ng TP, Carre C, Narang V, Wong G et al. Systemic and metabolic signature of sarcopenia in community-dwelling older adults. *The Journals of Gerontology: Series A*. 2020;75(2):309-17.

46. Demirdağ F, Kolbaşı EN, Aykut GB, Güler KY, Murat S, Oztürk GB et al. Nutritional status as a mediator between the age-related muscle loss and frailty in community-dwelling older adults. *Archives of Gerontology and Geriatrics*. 2022;98:104569.

47. Calcaterra L, van Kan GA, Steinmeyer Z, Angioni D, Proietti M, Sourdet S. Sarcopenia and poor nutritional status in older adults. *Clinical Nutrition*. 2024;43(3):701-7.

48. Vidaña-Espinoza HJ, López-Teros MT, Esparza-Romero J, Rosas-Carrasco O, Luna-López A, Alemán Mateo H. Association between the risk of malnutrition and sarcopenia at 4.2 years of follow-up in community-dwelling older adults. *Frontiers in Medicine*. 2024;11:1363977.

49. Gümüşsoy M, Atmış V, Yalçın A, Bahşi R, Yiğit S, Arı S et al. Malnutrition-sarcopenia syndrome and all-cause mortality in hospitalized older people. *Clinical Nutrition*. 2021;40(11):5475-81.

50. Hu X, Zhang L, Wang H, Hao Q, Dong B, Yang M. Malnutrition-sarcopenia syndrome predicts mortality in hospitalized older patients. *Scientific reports*. 2017;7(1):3171.

51. Macedo C, Amaral TF, Rodrigues J, Santin F, Avesani CM. Malnutrition and sarcopenia combined increases the risk for mortality in older adults on hemodialysis. *Frontiers in nutrition*. 2021;8:721941.

52. Sobestiansky S, Åberg AC, Cederholm T. Sarcopenia and malnutrition in relation to mortality in hospitalised patients in geriatric care–predictive validity of updated diagnoses. *Clinical Nutrition ESPEN*. 2021;45:442-8.

53. Sousa IM, Burgel CF, Silva FM, Fayh APT. Prognostic value of isolated sarcopenia or malnutrition–sarcopenia syndrome for clinical outcomes in hospitalized patients. *Nutrients*. 2022;14(11):2207.
